# Acceptor–Acceptor-Type Conjugated Polymers for Energy Level Modulation in Semiconducting Carbon Nanotube Transistors

**DOI:** 10.1021/acsami.5c21549

**Published:** 2025-12-28

**Authors:** You-Chen Chen, Megumi Matsuda, Yi-Hsuan Tung, Guo-Hao Jiang, Yu-Che Kan, Shang-Wen Su, Chien-Chung Shih, Tomoya Higashihara, Yan-Cheng Lin

**Affiliations:** † Department of Chemical Engineering, 34912 National Cheng Kung University, Tainan 70101, Taiwan; ‡ Department of Organic Materials Science, Graduate School of Organic Materials Science, Yamagata University, 4-3-16 Jonan, Yonezawa, Yamagata 992-8510, Japan; § Department of Chemical Engineering and Materials Engineering, 34883 National Yunlin University of Science and Technology, Yunlin 64002, Taiwan; ∥ Advanced Research Center for Green Materials Science and Technology, National Taiwan University, Taipei 10617, Taiwan

**Keywords:** naphthalene diimide, bis(thienyl)imide, conjugated polymers, single-walled carbon nanotubes, field-effect transistors

## Abstract

Numerous methods have been developed for sorting single-walled carbon nanotubes (SWCNTs), and polymer wrapping has become one of the most widely applied strategies for obtaining high-purity semiconducting SWCNTs (s-SWCNTs). Conjugated polymers can selectively sort s-SWCNTs, yet the role of frontier orbital level tuning in this process remains underexplored. In this study, we designed and compared four naphthalene diimide (NDI)–based copolymers with distinct backbones. Among them, the NDI-copolymers with a bithiophene donor (PNDI-2T) or a bis­(thienyl)­imide acceptor (PNDI-BTI) can effectively wrap onto the s-SWCNTs for investigating how modulation of the frontier energy levels affects polymer–nanotube interactions and device performance. Optical characterizations revealed that PNDI-BTI achieves s-SWCNT purity above 99%, higher than that obtained with PNDI-2T. Furthermore, PNDI-BTI exhibits stronger aggregation, leading to enhanced wrapping and the formation of longer, more uniform s-SWCNT networks, whereas PNDI-2T tends to produce more bundled structures. On the other hand, photoluminescence excitation mapping confirmed that the two polymers selectively wrap distinct nanotube chiralities, leading to noticeable differences in average tube diameter: PNDI-BTI favors large-diameter s-SWCNTs, while PNDI-2T preferentially interacts with small-diameter ones. Field-effect transistors fabricated with PNDI-BTI/s-SWCNTs show superior performance, exhibiting a high hole mobility of 2.1 cm^2^ V^–1^ s^–1^ and a stably *I*_on_/*I*_off_ ratio of 9 × 10^3^, as well as improved endurance and bias stability, which are attributed to the lower-lying energy levels and stronger π–π interactions of PNDI-BTI. These findings demonstrate that tuning the frontier orbital levels of polymers provides an effective strategy to improve sorting selectivity and device performance, offering new insights into the design of high-performance acceptor–acceptor type conjugated polymers for s-SWCNT sorting.

## Graphical abstract

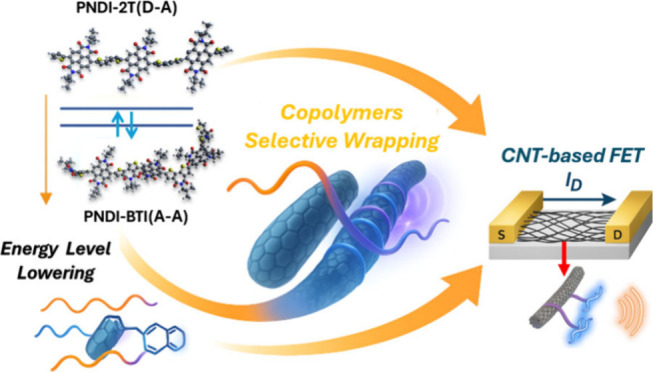

## Introduction

Single-walled carbon nanotubes (SWCNTs) have attracted tremendous interest as one of the most promising materials for microelectronics, including field-effect transistors (FETs),^1–3^ photovoltaic cells,^4–6^ and even light-emitting diodes.^7–9^ Their exceptional carrier mobility^10^ and mechanical flexibility^11^ endow them with remarkable electrical properties, making semiconducting SWCNTs (s-SWCNTs) particularly attractive for FET applications. s-SWCNTs enable transistors with steep subthreshold swings and low-voltage operation. At the same time, their unique nanoscale form factor not only facilitates dense device integration but also offers compatibility with scalable solution-based fabrication. Taken together, these attributes have positioned s-SWCNTs as strong contenders for next-generation electronics, and several reports have demonstrated that high-purity s-SWCNT FETs can even outperform state-of-the-art silicon devices at comparable channel lengths.^12^ However, SWCNTs typically contain a heterogeneous mixture of semiconducting and metallic nanotubes in an approximate 2:1 ratio. The residual metallic fraction forms unwanted conduction pathways that induce large off-state leakage currents (*I*_off_) and reduce the on/off current ratio (*I*_on_/*I*_off_), thereby limiting the use of SWCNTs in high-performance electronic devices. To address this issue, several postgrowth separation strategies have been developed, including (i) density-gradient ultracentrifugation^13,14^ and (ii) gel chromatography^15,16^ or electrophoresis.^17^ These methods have convincingly demonstrated that high-purity s-SWCNTs can be obtained. Still, their inherent limitations in scalability, cost, and processing efficiency prevent widespread application, thereby motivating the development of more practical, scalable alternatives.

To address these challenges, conjugated polymer-assisted sorting has emerged as a promising strategy for producing high-purity s-SWCNTs. Among them, polyfluorene derivatives (PFs)^18–20^ were the earliest and most extensively applied class. Nish et al. first demonstrated in 2007 that PFs could successfully achieve selective dispersion.^21^ Subsequent studies further revealed that varying the alkyl side-chain length of PFs allows selective dispersion across different diameter ranges of s-SWCNTs, producing high-purity dispersions that enabled nanotube network transistors with competitive carrier mobility (μ) and *I*_on_/*I*_off_.^22–24^ On the other hand, polythiophene-based polymers^25,26^ have also been widely explored. Lee et al. demonstrated that regioregular poly­(3-alkylthiophene) can effectively sort larger-diameter nanotubes and yield high-purity dispersions.^27^ However, polythiophenes with relatively wide bandgaps suffer from poor energy-level alignment with s-SWCNTs, often introducing interfacial barriers and significant charge trapping that increase *I*off, thereby limiting their utility in high-performance devices.^28^ Moreover, because p-type polymers interact strongly with nanotube surfaces, residual polymers are often difficult to remove after sorting, thereby causing variability in device performance.^22^

To overcome the limitations of early all-donor-type polymers, recent studies have turned to donor–acceptor (D–A) backbones. Lei et al. have shown that low-bandgap diketopyrrolopyrrole (DPP)-based polymers can selectively disperse semiconducting nanotubes with higher efficiency, boosting both yield and selectivity and leading to high-performing transistors.^29^ Following this, naphthalene diimide (NDI) derivatives emerged as another critical class of D–A polymers.^30,31^ More recent work has even explored extended conjugation in the side-chain domain to suppress aggregation and further enhance wrapping. In previous studies, most high-performance conjugated polymers for carbon nanotube sorting and FET applications have been designed with D–A type backbones. However, little attention has been given to acceptor–acceptor (A–A) type polymers. Compared with conventional D–A-type polymers, A–A backbones offer distinct advantages by enabling a more pronounced lowering of the frontier orbital energy levels. For instance, replacing donor units with strong acceptor moieties can effectively decrease the LUMO,^32^ thereby stabilizing electrons against oxygen or moisture trapping.^33^ Moreover, a lower LUMO helps suppress *I*_off_, thereby improving the *I*_on_/*I*_off_ ratio and enabling more reliable switching behavior. Moreover, most reports have primarily focused on the separation between metallic and semiconducting species. Far fewer investigations have discussed the chirality selectivity. The electronic band structure of SWCNTs is governed by chirality (n, m). The chirality of the SWCNT dictates the optical transitions and charge-transport characteristics that underpin device operation.

In this study, we designed and compared four NDI-based conjugated polymers with distinct backbone structures: poly­(naphthalene diimide-*alt*-bis­(thienyl)­imide) (PNDI-BTI), poly­(naphthalene diimide-*alt*-biselenophene) (PNDI-2Se), and poly­(naphthalene diimide-*alt*-bisthiazole) (PNDI-2Tz), A–A type polymers with deeper frontier orbital levels, and poly­(naphthalene diimide-*alt*-bithiophene) (PNDI-2T), a conventional D–A type analogue. These polymers were employed to wrap and selectively sort plasma-discharge (PD) SWCNTs, to investigate how energy-level modulation governs sorting efficiency and device characteristics. We first employed molecular dynamics (MD) simulations to examine interactions between polymers and SWCNTs, revealing that PNDI-BTI forms a more stable binding conformation on the nanotube surface. To validate this prediction, optical characterizations including UV–vis–NIR absorption, Raman spectroscopy, and photoluminescence emission (PLE) mapping were used. The results confirmed that PNDI-BTI can more effectively enrich s-SWCNTs and preferentially sort larger-diameter s-SWCNTs, whereas PNDI-2T exhibited relatively weaker selectivity. Subsequent morphological analysis using atomic force microscopy (AFM) further revealed that CNTs dispersed by PNDI-BTI were more uniformly distributed, with longer tube lengths and larger diameters. Finally, the sorted s-SWCNT hybrids were fabricated into FET devices and evaluated electrically. The devices based on PNDI-BTI demonstrated superior performance, characterized by higher μ and *I*on/*I*off, and a lower threshold voltage. These findings indicate that the A–A type backbone facilitates a favorable conformation for sorting s-SWCNT and better energy-level alignment with s-SWCNT, thereby improving FET device performance.

## Results and Discussion

### Molecular Design and Characterization of the Polymers

The conjugated polymers were synthesized through Stille cross-coupling polymerization with dibromo NDI monomers and distannyl comonomers. The syntheses and chemical structure characterizations of monomers and polymers are presented in Figures S1–S5 and detailed in the Supporting Information. Conjugated polymers have been widely studied for the selective separation of s-SWCNTs. In the process, the polymers wrap around the nanotube surfaces, enabling the extraction of high-purity s-SWCNTs that can be directly integrated into FET devices. In this study, we designed two NDI-based copolymers to investigate how energy levels affect nanotube selectivity and device performance. As shown in Figure 1a, PNDI-BTI, PNDI-2Se, and PNDI-2Tz adopt A–A structures, which lowers the LUMO level through its strong electron-deficient effect. In contrast, PNDI-2T is a D–A copolymer incorporating a bithiophene unit, resulting in a higher LUMO and a more planar backbone. For device fabrication, the sorted polymer/SWCNT hybrids were transferred onto Si/SiO_2_ substrates (300 nm). A thin SBS dielectric (∼30 nm) was coated (as summarized in Table S1), and Au contact electrodes were deposited through a shadow mask. In the resulting bottom-gate, top-contact structure (Figure 1b), the polymer-wrapped SWCNT network serves as the semiconducting channel, with a thickness of approximately 10 nm. Figure 1c and Figure S7 (Supporting Information) show the aggregation behavior of the PNDI-BTI and PNDI-2T. The calculation is detailed in the Experimental Section. The aggregation fraction of PNDI-BTI is approximately 0.60, which is significantly higher than that of PNDI-2T (0.38), as shown in Figure 1d. Previous studies have revealed that stronger polymer aggregation generally reduces the overall dispersion yield but enhances chiral selectivity by promoting more specific π–π interactions with nanotube surfaces.^34^ In our study, stronger aggregation of PNDI-BTI indeed facilitates the selective stabilization of specific SWCNT species, whereas weaker aggregation of PNDI-2T yields higher dispersion but sacrifices chiral discrimination. These contrasting behaviors are consistent with our sorting results, further confirming the correlation between aggregation tendency and chiral selectivity. To further examine the aggregation behavior of the conjugated polymers, photoluminescence excitation (PLE) measurements were performed on the pure polymer solutions, as shown in Figure 1e. A pronounced contrast is observed between PNDI-BTI and PNDI-2T. While PNDI-2T exhibits a broad and detectable fluorescence response over a wide excitation–emission range, the PNDI-BTI polymer shows strongly suppressed emission across the entire spectral window, resulting in an almost featureless PLE map. Such fluorescence suppression is commonly associated with enhanced intermolecular interactions in aggregated conjugated polymer systems, in which excited states preferentially relax nonradiatively rather than emit photons. From this perspective, the PLE results suggest that PNDI-BTI is more prone to aggregate in solution than PNDI-2T.

**Figure 1:**
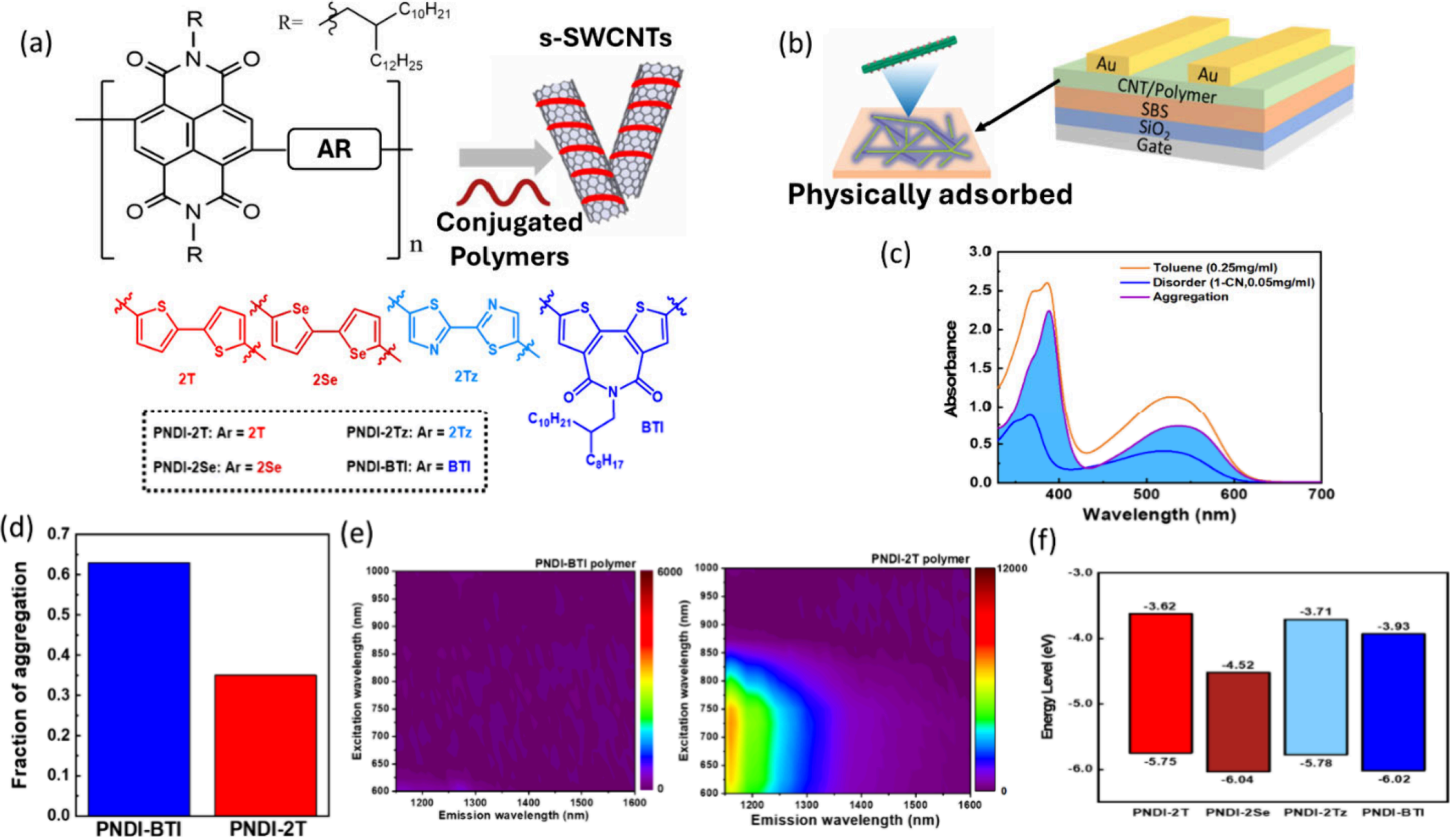
(a) Chemical structure of the NDI-based conjugated polymers for the s-SWCNT sorting. (b) FET device structure with a semiconducting channel composed of polymer/s-SWCNT and the bilayered insulating dielectrics comprising a 30 nm-thick cross-linked SBS layer and a 300 nm-thick SiO_2_ on a highly n-doped silicon wafer. (c) Fraction of aggregate and disorder observed from the UV–vis–NIR absorption spectra of PNDI-BTI in the solutions. (d) The fraction of aggregation of the polymer solutions. (e) Photoluminescence excitation (PLE) maps of the polymer solutions in toluene at a concentration of 0.25 mg mL^–1^: PNDI-BTI (left) and PNDI-2T (right). (f) Frontier energy levels of the conjugated polymers.

Figure 1f, Figure S6, and Table S2 (Supporting Information) present the energy level of the polymer calculated by UV–vis–NIR spectroscopy. The lowest unoccupied molecular orbital (LUMO) level was estimated from cyclic voltammetry (CV, Figures S8 and S9, Supporting Information) according to LUMO = −e­(*E*_red_ – *E*_1/2(Fc/Fc^+^)_ + 4.8), where *E*_red_ is the onset reduction potential and *E*_1/2(Fc/Fc^+^)_ is the half-wave potential of ferrocene. The optical bandgap (*E*_g_) was obtained from the absorption onset using the relation *E*g = 1240/λ_onset_. The highest occupied molecular orbital (HOMO) level was then determined by subtracting the bandgap from the LUMO (HOMO = LUMO – *E*_g_). From the calculations, the LUMO and HOMO levels of PNDI-BTI were determined to be −3.93 and −6.02 eV, respectively, whereas those of PNDI-2T were −3.62 and −5.75 eV, respectively. The lower LUMO level of PNDI-BTI, PNDI-2Se, and PNDI-2Tz can be attributed to their A–A configurations, in which the bis­(thienyl)­imide, biselenophene, and bithiazole unit provides strong electron-withdrawing effects that stabilize the conduction band. Such electron-deficient systems exhibit reduced electron density and lower-lying LUMO levels, which enhance electronic affinity and enable weak charge-transfer with the band-edge states of s-SWCNTs. Previous studies on diimide-based acceptor motifs, such as NDI and perylene diimide (PDI), have demonstrated that this type of electronic complementarity can increase adsorption energy and stabilize s-SWCNTs over electron-rich polymers.^35^ In addition to electronic effects, selective polymer–SWCNT association is also influenced by geometric compatibility between the polymer backbone and the nanotube surface.^22^ As a result, for the electron-deficient A–A type NDI polymers investigated in this work, low-LUMO-driven electronic affinity and favorable interfacial packing act cooperatively to govern their interaction with s-SWCNTs, ultimately impacting sorting selectivity and device performance.

### Sorting Characteristics of the Polymer/s-SWCNTs

Polymer/s-SWCNT sorting was achieved via ultrasonic dispersion of raw SWCNTs with conjugated polymers in toluene. Sonication enabled polymer backbones to selectively adsorb onto nanotube surfaces. Subsequent centrifugation removed bundles, m-SWCNTs, and amorphous carbon. UV–vis–NIR absorption spectroscopy was used to examine the optical signatures of the sorted solutions, as shown in Figure 2a–d for PNDI-BTI, PNDI-2Se, PNDI-2Tz, and PNDI-2T, respectively. In the UV–vis–NIR absorption spectra, two characteristic regions can be identified: the *S*_22_ and *S*_11_ transitions, further confirming the presence of semiconducting species. Furthermore, two crucial parameters were defined: selectivity (ϕ) and yield. The purity of SWCNTs is determined by ϕ, defined as *A*_S22_/(*A*_S22_ + *A*_baseline_),^36^ where *A*_S22_ is the integral of the *S*_22_ peak and *A*baseline is the integral of the baseline. The yield of s-SWCNTs was estimated based on Beer’s law (*A* = *εbc*), where c represents the concentration of sorted s-SWNTs and *ε* is the absorption coefficient. After determining the concentration, the yield can be expressed as Yield = (*C*_s‑SWCNTs_ × *V*_sorting_)/(2*W*_SWNTs_/3), where *C*s-SWCNT is the concentration of s-SWCNTs in the supernatant, *V*_sorting_ represents the total volume of the sorted solution, and WSWNT is the initial mass of SWCNTs used for sorting. The factor of 2/3 accounts for the fraction of semiconducting tubes in SWCNT mixtures. The sorting parameters are summarized in Table S3 (Supporting Information). Based on these calculations, the ϕ values of PNDI-BTI and PNDI-2T were determined to be 0.47 and 0.22. From the literature, higher ϕ values indicate higher s-SWCNTs purity, and ϕ values >0.40 were correlated with purity >99%.^37,38^ The yields were further estimated to be 18.1% for PNDI-BTI and 48.8% for PNDI-2T. These results suggest that the larger A–A configuration of PNDI-BTI may induce a less coplanar backbone, promoting stronger selectivity toward semiconducting nanotubes but limiting overall wrapping efficiency, thereby lowering yield. In contrast, the more planar D–A structure of PNDI-2T is likely to wrap broader nanotube coverage, resulting in a higher yield but lower selectivity. PNDI-2Se and PNDI-2Tz show poor ability to wrap around s-SWCNTs and are therefore excluded from subsequent characterizations of the polymer/SWCNT systems.

**Figure 2:**
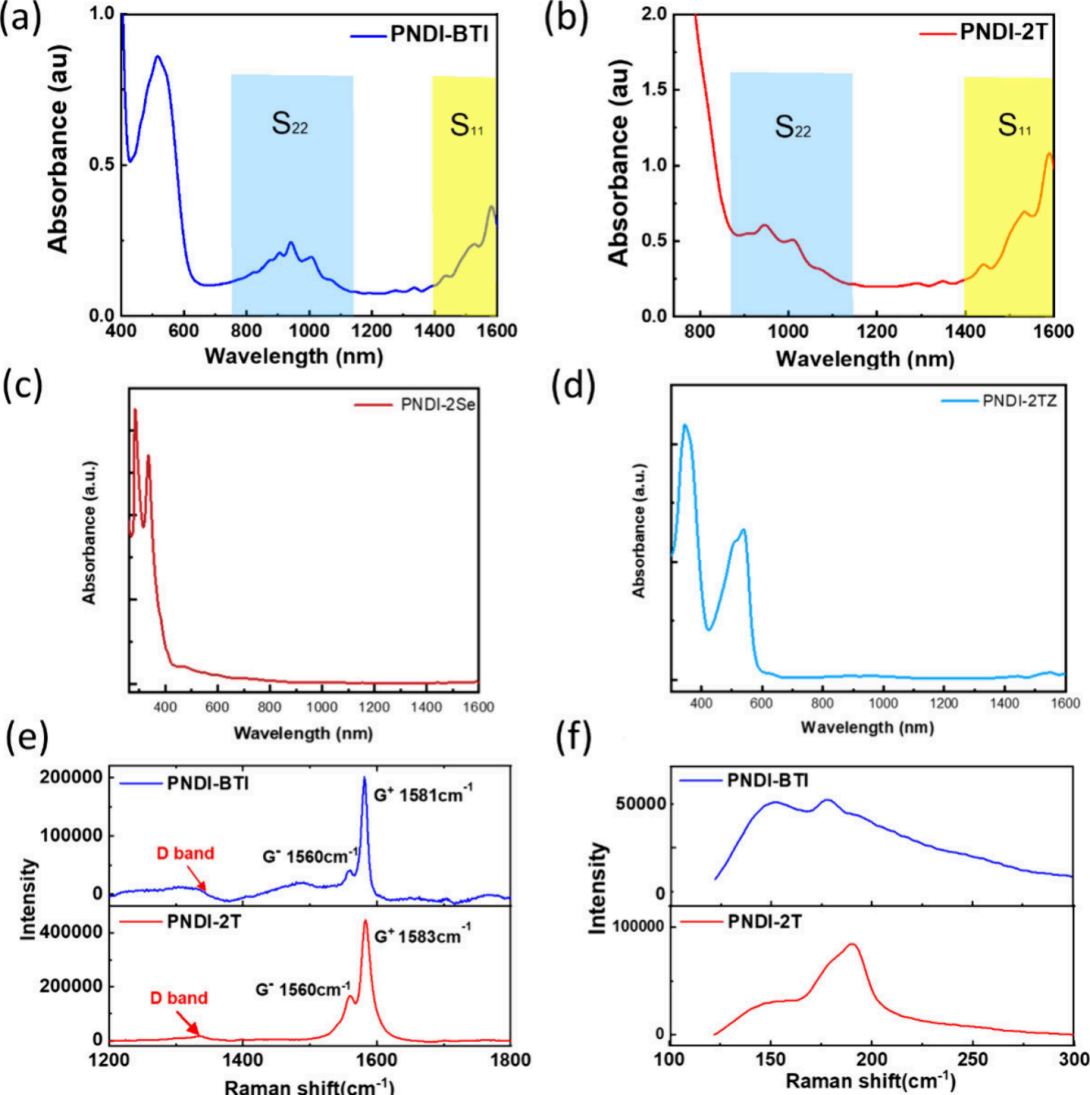
Optical absorption spectra of the polymer/s-SWCNT sorting solutions with (a) PNDI-BTI, (b) PNDI-2T, (c) PNDI-2Se, and (d) PNDI-2Tz. Raman spectra of drop-cast polymer/s-SWCNT films with an excitation wavelength of 633 nm at the bands spanning the range of (e) 1200–1800 cm^–1^ region and (f) 100–300 cm^–1^ for PNDI-BTI (top) and PNDI-2T (bottom).

To further examine the structural integrity and purity of the sorted s-SWCNTs, Raman spectroscopy with 633 nm laser excitation was performed on drop-cast films prepared from the polymer/SWCNT dispersions. Figure 2e and f displays the Raman spectra’s high-wavenumber and low-wavenumber regions. In the high-wavenumber Raman region, the G band can be divided into two components: the G^+^ peak at ∼1590 cm^–1^ and the G^–^ peak at ∼1570 cm^–1^. Figure S10 presents the Raman spectra of pure polymer films. No polymer signal is observed in the spectra of the polymer/SWCNT films. This result elicits that the rinsing process has thoroughly removed the polymer. In particular, PNDI-BTI exhibits a higher G^+^/G^–^ intensity ratio (4.16) than PNDI-2T (2.64). As reported in previous studies, the line shape and relative intensity of the G-band are closely related to the electronic character of SWCNTs, particularly the distinction between s-SWCNTs and m-SWCNTs.^39^ In this regard, the higher G^+^/G^–^ ratio observed for PNDI-BTI is consistent with a more pronounced semiconducting-enriched character after polymer sorting. The D band (∼1350 cm^–1^) originates, and the G/D intensity ratio is commonly adopted as a measure of defect density in SWNTs.^40^ In our study, PNDI-BTI and PNDI-2T exhibited G/D ratios of 20.8 and 27.5, both substantially higher than the ∼9.2 reported for raw SWNTs in a previous report from Su et al.^41^ The improvement relative to pristine samples confirms that polymer-assisted sorting not only enriches semiconducting nanotubes but also effectively removes defective and metallic species. Next, in the low-wavenumber region (100–300 cm^–1^), distinct radial breathing mode (RBM) features were observed (Figure 2f). The RBM frequency is inversely proportional to nanotube diameter (ω_RBM_ ≈ *A*/dt + *B*, with *A* ≈ 234 cm^–1^·nm and *B* ≈ 10 cm^–1^).^42^ For PNDI-BTI, RBM peaks at 176 and 150 cm^–1^ correspond to diameters of approximately 1.41 and 1.67 nm, indicating a preference for medium-to-large diameter nanotubes. In contrast, PNDI-2T exhibited a single RBM peak at 192 cm^–1^, corresponding to a smaller diameter of ∼1.29 nm. This difference suggests that PNDI-BTI shows greater selectivity for midsized s-SWCNTs, whereas PNDI-2T captures a smaller-diameter population.

### Photoluminescence Excitation Characterization of the Polymer/s-SWCNT Solutions

To further investigate the chiral selectivity of the polymer/SWCNT dispersions, PLE spectroscopy was employed. In the PLE map, each bright spot corresponds to the emission of the *S*11 transition when the *S*22 excitation is resonantly matched, allowing the assignment of distinct (n, m) chiral species of s-SWCNTs. By referencing established (n, m) chirality maps, these observed species can be classified as semiconducting or metallic SWCNTs.^43,44^ As shown in Figure 3a and b, both PNDI-BTI and PNDI-2T successfully enriched semiconducting nanotubes, but apparent differences in their chiral distributions are evident. Importantly, the enriched peaks in both samples are predominantly assigned to semiconducting tubes, further confirming that the conjugated polymers drive preferential extraction of s-SWCNTs. To further quantify the meaning of chiral distribution, we convert the PLE intensities into nanotube diameters and calculate the relative abundance of different chiral species (as shown in Figure 3c).^45^ In the PNDI-BTI sample, the enriched nanotubes are mainly concentrated in the medium-to-large diameter range (1.23–1.36 nm), with representative chiralities such as (11,7), (10,9), (12,7), and (15,4). By contrast, PNDI-2T shows a stronger tendency toward smaller-diameter nanotubes (<1.1 nm), including (12,1), (10,5), and (11,3), in agreement with the Raman spectroscopy results, demonstrating again the PNDI-BTI’s increased wrapping/sorting ability for midto-larger-diameter tubes. We propose that this difference arises because the less coplanar backbone of PNDI-BTI allows localized π–π stacking to better match the curvature of larger-diameter nanotubes, thereby providing stronger selectivity for these species. In contrast, the more planar backbone of 2T enables broader wrapping interactions, making it easier to capture smaller-diameter nanotubes and leading to a broader overall distribution.

**Figure 3:**
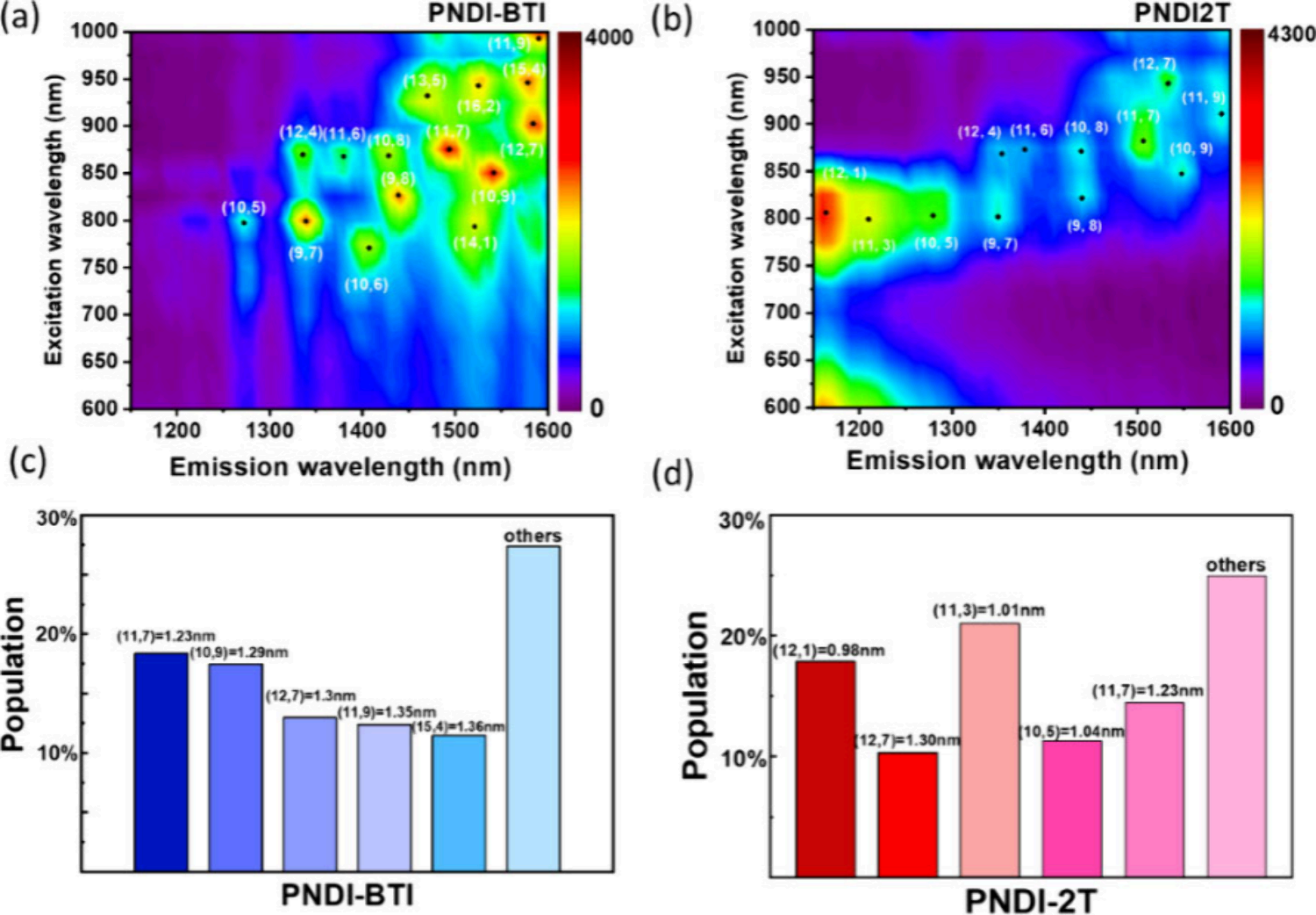
(a, b) 2D PLE maps and (c, d) the leading chirality populations and the corresponding diameter distributions of the polymer/s-SWCNT sorting solutions with (a, c) PNDI-BTI and (b, d) PNDI-2T. Note that the solutions are with a polymer concentration of 0.25 mg mL^–1^ in toluene.

### Molecular-Level Insights into Polymer/s-SWCNT Interactions

To quantify the backbone planarity of the polymers, density functional theory (DFT) calculations were performed using trimer models of PNDI-BTI, PNDI-2T, PNDI-2Tz and PNDI-2Se. The optimized geometries revealed apparent torsional differences. For PNDI-BTI, the dihedral angle between two acceptor units (NDI and BTI) was about 80–110°, showing a highly twisted backbone. In contrast, PNDI-2T exhibited a more planar structure, with the NDI–thiophene angle of approximately 60° and the thiophene–thiophene angle of approximately 10°. PNDI-2Se shows an even higher degree of coplanarity, with dihedral angles concentrated in the 30–40° range, whereas PNDI-2Tz displays an uneven distortion comprising a highly distorted NDI–thiazole twists exceeding 100° along the main chain and a coplanar thiazole–thiazole linking (2–3°). This disparity will make it difficult to wrap the polymer stably around nanotubes. These results indicate that PNDI-BTI and PNDI-2T adopt an intermediate torsional regime that balances backbone flexibility and π-conjugation, whereas PNDI-2Se is overly planar and rigid, and PNDI-2Tz suffers from excessive torsional distortion that disrupts conjugation continuity. Consistent with this interpretation, PNDI-BTI exhibits a higher aggregation fraction in solution and achieves higher purity in the sorted s-SWCNTs, while PNDI-2T shows moderate selectivity, and PNDI-2Se and PNDI-2Tz display inferior sorting performance. MD simulations can capture the complex interactions within polymer/s-SWCNTs and more realistically analyze absorption energies. In our model, PNDI-BTI and PNDI-2T chains containing 10 repeat units were placed in contact with a (10, 9) s-SWCNT, which served as the representative nanotube. From the simulation snapshots shown in Figure 4e and f, both polymers were found to adsorb onto the nanotube surface primarily through π–π interactions between the conjugated backbones and the graphitic wall. To further quantify the interaction strength between the polymers and s-SWCNTs, the binding energies (*E*b) were calculated from the MD-equilibrated structures. The details of the calculations are shown in the Experimental Section. The PNDI-BTI system showed a more negative *E*b of −232 kcal/mol, which is much larger in magnitude than that of PNDI-2T (−106 kcal/mol), indicating a more stable interaction with the nanotube surface. This stronger binding can be attributed to the less planar backbone and larger acceptor units of PNDI-BTI, which enable it to wrap more tightly and adopt a more stable configuration around the nanotubes. Such stability helps explain why BTI tends to preferentially sort s-SWCNTs with higher purity, thereby contributing to improved device performance.

**Figure 4:**
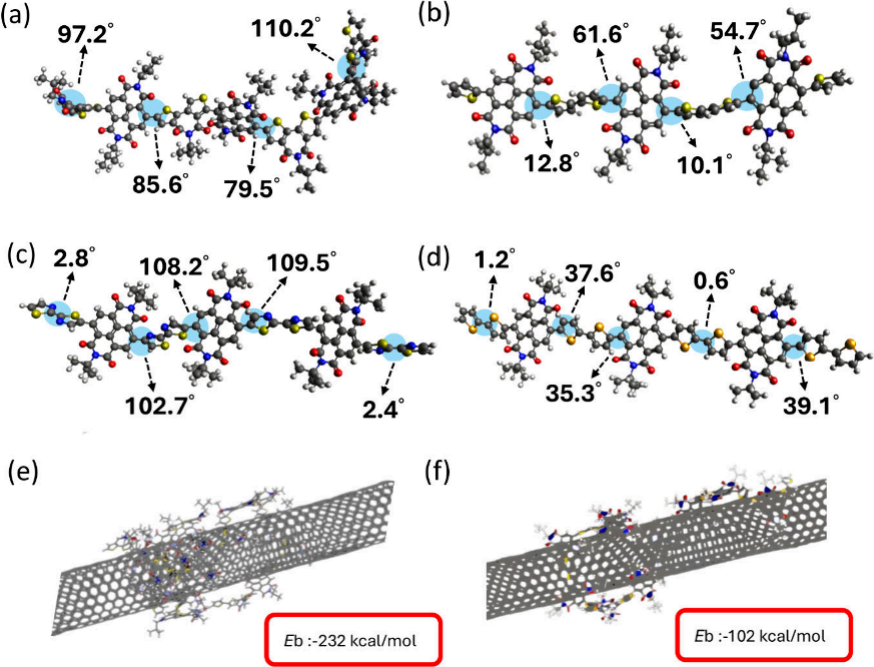
Optimized molecular structures of polymers simulated from DFT calculations: (a) PNDI-BTI, (b) PNDI-2T, (c) PNDI-2Tz, and (d) PNDI-2Se. MD simulated conformation and binding energy of the polymer/s-SWCNT for (e) PNDI-BTI/s-SWCNT and (f) PNDI-2T/s-SWCNT.

### Morphological Analysis of Polymer/SWNT Films

Before fabricating FET devices, the morphology of the polymer/s-SWCNT thin films was examined by atomic force microscopy (AFM). This technique provides a clear view of how nanotubes are dispersed on the substrate and whether they form aggregated or bundled structures. The AFM images also help evaluate how these morphological differences may influence charge transport in the devices. Figure 5a and d shows the AFM images of SWNTs wrapped with PNDI-BTI and PNDI-2T, the right side mapping technique extracted from the GTFiber program developed by Persson et al.^46^ The setting parameter of the software is shown in Figure S11 (Supporting Information). As shown, the PNDI-BTI film exhibits a smoother surface, weaker height contrast, and more uniformly distributed nanotubes. In contrast, the PNDI-2T/s-SWCNT film exhibits greater brightness variations and more pronounced surface corrugations, resulting in higher roughness. In addition to AFM, SEM images included in Figure Figure 5a and d provides complementary information on the nanotube network morphology. The SEM results further support the difference in nanotube network density between the two polymer systems, with the PNDI-BTI film forming a denser network than the PNDI-2T system. These observations are consistent with the trends observed in the electrical characteristics of the corresponding devices. Comparing AFM and SEM images, Mirka et al. report that although polymers remain on the s-SWCNT surface after solvent rinsing, these residues play a trivial role in device performance.^47^ The coverage and nanotube dimension play more critical roles. Accordingly, Figure 5b and e shows the nanotube length distributions for PNDI-BTI and PNDI-2T films, respectively. The PNDI-BTI-wrapped samples exhibit a longer average nanotube length (252 nm) than the 2T samples (210 nm), although both display relatively broad distributions. It can be reasonably inferred that the longer nanotubes in the PNDI-BTI films facilitate the formation of denser, more interconnected networks, which, in turn, provide more continuous charge-transport pathways. In addition, the stable wrapping of PNDI-BTI around the nanotubes may further improve the electrical performance of the resulting devices. Next, Figure 5c and f shows the nanotube width distributions of the two films. The BTI samples have a larger average width (27.8 nm) than the 2T samples (24.7 nm). These results suggest that the more extended A–A backbone of PNDI-BTI preferentially wraps nanotubes with larger apparent diameters, consistent with its stronger intermolecular interactions and greater wrapping ability. However, the topographic diameters measured by AFM are typically higher than those obtained from MD simulations (∼3 nm with polymer wrapping), PLE (1.0–1.4 nm), or Raman spectroscopy (1.3–1.7 nm) due to the following aspects (i) AFM’s limited topographic resolution, (ii) multilayer polymer wrapping on the surface, and (iii) the systematic differences among the characterization methods. Nevertheless, the relative comparison still clearly indicates that PNDI-BTI prefers nanotubes with larger diameters and longer lengths than those sorted from PNDI-2T, and these advantages can lead to better charge-transport performance in FET applications.

**Figure 5:**
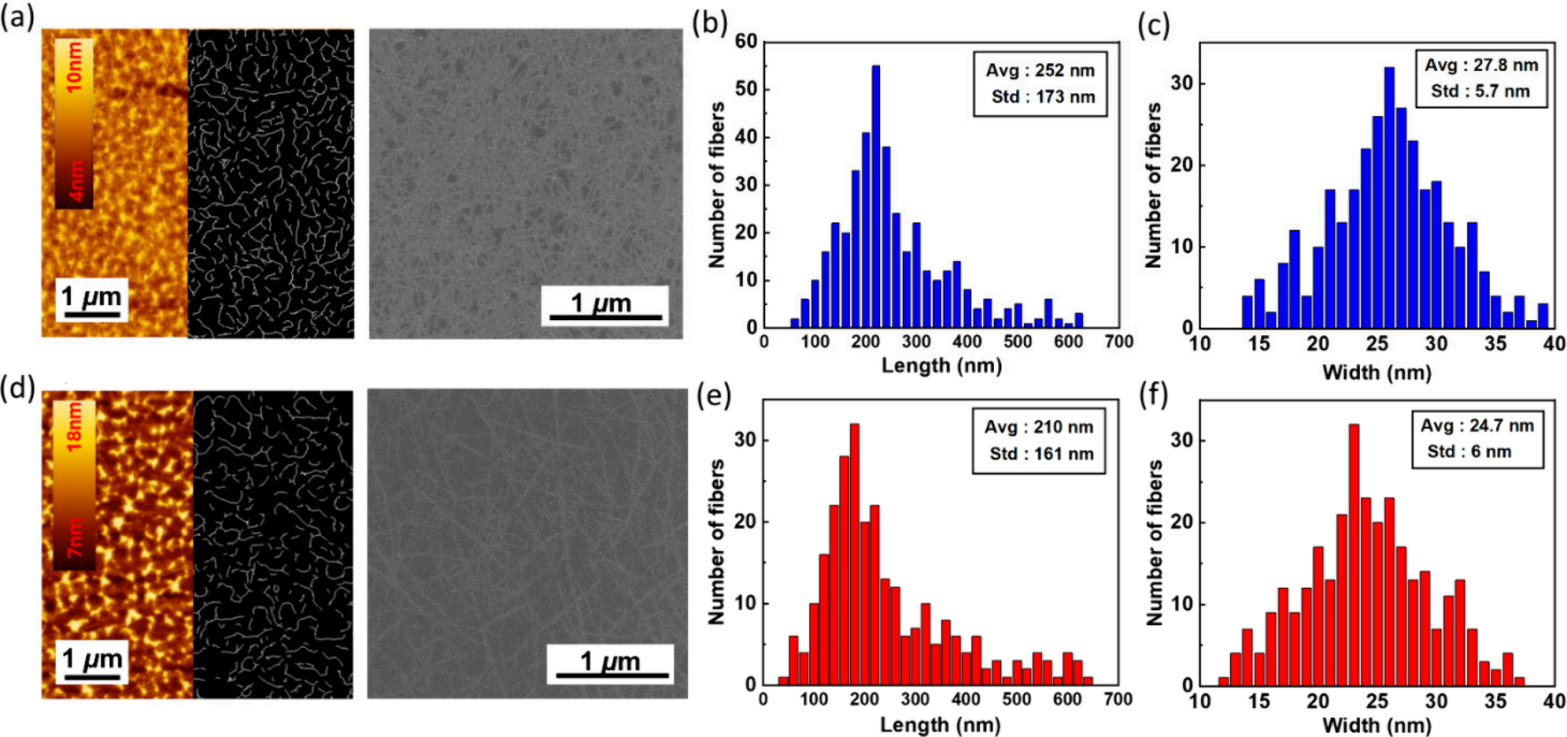
(a, d) AFM (left) and SEM (right) images of polymer/s-SWCNT hybrid films, (b, e) the length distributions, and (c, f) the diameter distributions of the s-SWCNT extracted by AFM topographic image mapping analysis using GT Fiber. The conjugated polymers applied to the sorting are (a–c) PNDI-BTI and (b–d) PNDI-2T. Note that the cropped AFM height images reveal the original topographies (left) and the image maps (right) in the same dimensional scale of 5 μm × 5 μm.

### Electrical Characterization of FET Devices

After analyzing the morphology of the polymer/s-SWCNT films, we next tested their FET device performance. The polymer/s-SWCNT networks were transferred onto Si/SiO_2_ substrates coated with a cross-linked SBS dielectric layer (30 nm). Gold electrodes were then thermally evaporated through a shadow mask to form the source and drain contacts, giving bottom-gate/top-contact (BG/TC) FET devices, as illustrated in Figure 1b. More detailed fabrication steps are described in the Experimental Section. As expected from the fundamental properties of s-SWCNTs, both PNDI-BTI and PNDI-2T devices showed typical p-type transfer behavior. The transfer characteristics of the polymer/s-SWCNT FET devices are shown in Figure 6a and b recorded by sweeping the gate voltage (*V*_g_) from 20 to −60 V, and the corresponding hole mobility (μ), threshold voltage (*V*_th_), and on/off current are summarized in Table S4 (Supporting Information). At a low drain bias (*V*_d_ = −10 V), the PNDI-BTI devices showed a mobility of 0.83 cm^2^ V^–1^ s^–1^, which is more than four times that of the PNDI-2T devices. (0.21 cm^2^ V^–1^ s^–1^). Moreover, when the drain bias was increased (*V*_d_ = −100 V, Figure S12, Supporting Information), the μ further increased to 2.11 cm^2^ V^–1^ s^–1^ for PNDI-BTI and 0.61 cm^2^ V^–1^ s^–1^ for PNDI-2T, demonstrating the consistent advantage of PNDI-BTI across different operating conditions. This improvement can be reasonably explained by the structural and spectroscopic analyses discussed earlier.

**Figure 6:**
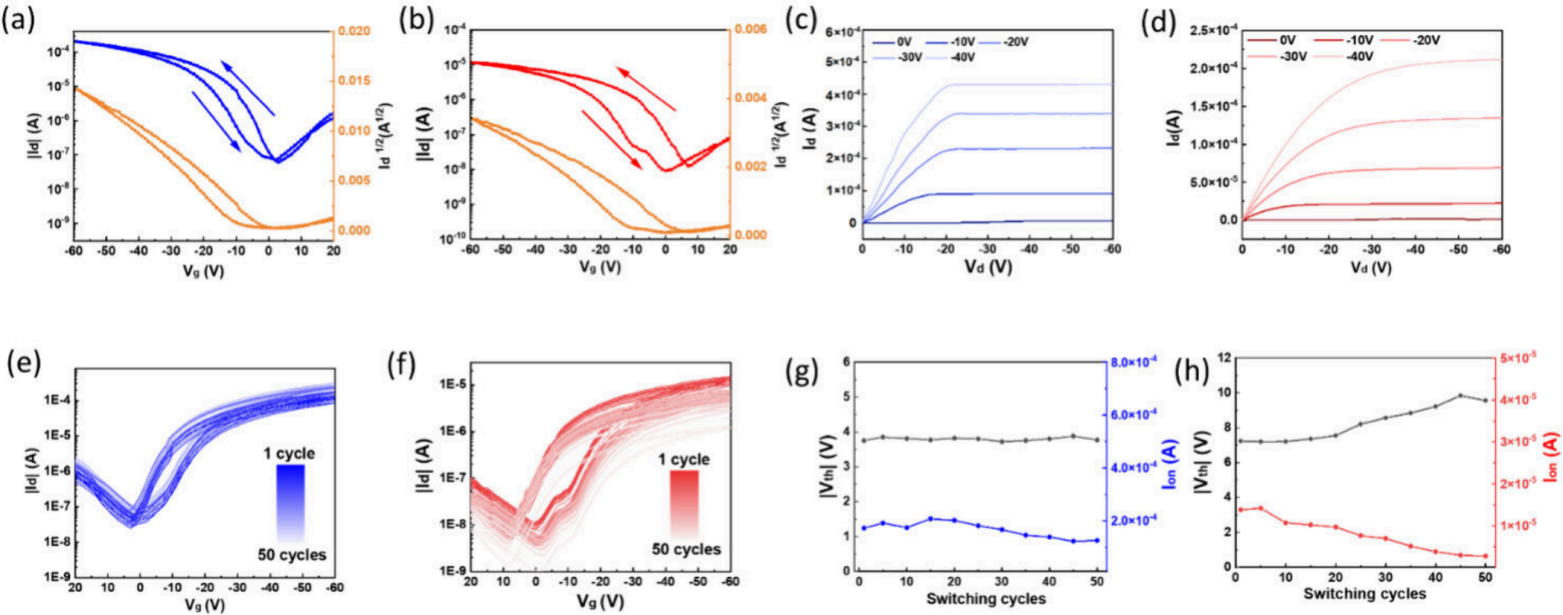
(a,b) Transfer, (c, d) output, (e, f) 50 consecutive transfer characteristics, and (g, h) the corresponding *V*_th_ and *I*_on_ variations along the switching cycles of the FET devices comprising the polymer/s-SWCNT hybrid films with (a, c, e, g) PNDI-BTI and (b, d, f, h) PNDI-2T. Note that the transfer curve was forwardly swept from 20 to −60 V at *V*_d_ = −10 V.

AFM revealed that the PNDI-BTI/s-SWCNT films contained longer nanotubes with slightly larger apparent diameters, which promote the formation of continuous conductive pathways. Raman spectroscopy indicated a lower defect density in the PNDI-BTI samples, reducing scattering sites that hinder carrier transport. In addition, the optical absorption spectra indicated higher s-SWCNT purity, thereby suppressing the influence of m-SWCNTs. In addition to the mobility, the *I*_on_/*I*_off_ also showed a clear difference between the two systems. As summarized in Table S4 (Supporting Information), the PNDI-BTI/s-SWCNT devices reached values of about 9 × 10^3^, whereas the PNDI-2T-based devices were limited to roughly 2 × 10^3^. This enhancement can be attributed to the lower energy levels of PNDI-BTI, which reduce the influence of trapping states on hole transport. As a result, charge carriers are more effectively removed during the off state, leading to a much lower *I*_off_ and a significantly higher *I*_on_/*I*_off_.

The output characteristics of the devices are shown in Figure 6c and d. For the PNDI-BTI/s-SWCNT devices (Figure 6c), the *I*_d_ increases steadily as the scaled-up *V*_g_. It reaches saturation rapidly at relatively low *V*_d_, with saturation currents significantly higher than those of the PNDI-2T/s-SWCNT devices.^48^ PNDI-2T devices (Figure 6d) show weaker current saturation and lower saturation currents. These differences highlight the improved saturation behavior and higher output currents of the PNDI-BTI devices, further confirming their superior charge-transport efficiency relative to PNDI-2T. Finally, the operational stability of the devices was evaluated by repeatedly measuring the transfer characteristics over 50 consecutive cycles. As shown in Figure 6e and f, the PNDI-BTI device maintained nearly identical transfer profiles even after 50 consecutive scans and exhibited slightly improved stability with reduced off-state noise. To further quantify stability during repeated operation, Figure 6g and h presents the evolution of *V*_th_ and *I*_on_ over the 50-cycle measurement. The PNDI-BTI device shows only minor *V*_th_ variation and a stable *I*_on_ across all cycles, confirming its strong resistance to performance changes under repeated bias. In contrast, the PNDI-2T device exhibits greater *V*_th_ drift and a gradual decrease in *I*_on_, indicating lower stability under continuous operation.

### Comparison of the Structure–Mobility Relationship of the Conjugated Polymer/s-SWCNT Hybrids

To gain a more comprehensive understanding of the mobility differences between the PNDI-BTI and PNDI-2T systems, we conducted an integrated comparison of several key parameters, including electrical characteristics, nanotube structural features, polymer–CNT interfacial indicators, and energetic alignment. Figure 7a summarizes the key electrical parameters and their relationship to the mobility trend. The mobility ratio (BTI/2T ≈ 4) is the most pronounced difference between the two systems and therefore serves as a reference point for examining other device metrics. The *I*_on_/*I*_off_ ratio also increases in BTI-based devices, and *V*th is slightly lower, which is generally beneficial for transistor operation. Although the increase in the *I*_on_/*I*_off_ ratio and the reduced *V*_th_ both follow the same favorable trend as the mobility enhancement, we believe that additional factors also contribute to the overall improvement in mobility. Based on the study by Zhou et al., presenting the square of the nanotube diameter as *D* ^2^ provides a more accurate description of its influence on electronic behavior.^38^ Therefore, in Figure 7b, we compare the diameters obtained from Raman, AFM, and PLE with the corresponding mobility values. In addition, the nanotube length (*L*) estimated from AFM is included to examine further whether geometric characteristics correlate with device performance. Both *D* ^2^ and *L* are positively correlated with mobility. The longer nanotubes reduce the number of tube–tube junctions, while larger s-SWCNTw possess smaller bandgaps and fewer defects, both of which contribute to enhanced charge transport. Figure 7c compares several Raman-derived parameters commonly used to characterize polymer–CNT interactions. Among these indicators, G^+^/G^–^ and ϕ show trends that are more consistent with the mobility enhancement observed in the PNDI-BTI system. In contrast, the G/D ratio differs only slightly between the two polymers. These results suggest that specific interfacial indicators are more closely associated with mobility enhancement, while others show weaker or negligible correlation. Figure 7d compares the energy-level parameters of the polymers and the s-SWCNT. As intended by the molecular design, energy-level tuning remains an essential factor governing electron injection and transport in polymer/s-SWCNT hybrid systems. The LUMO level of PNDI-BTI lies below that of the CNT, providing a more favorable energetic condition for electron injection, which is consistent with its higher mobility performance. However, it can be inferred that the additional mobility enhancement observed in the PNDI-BTI devices is not solely governed by energy-level alignment. The results reflect the cooperative influence of structural and interfacial factors built upon the foundation established by energy-level tuning. In other words, energy-level alignment provides a necessary condition for efficient charge transport, whereas actual electrical performance reflects the combined effects of multiple factors, including nanotube dimensions, purity, and defects.

**Figure 7:**
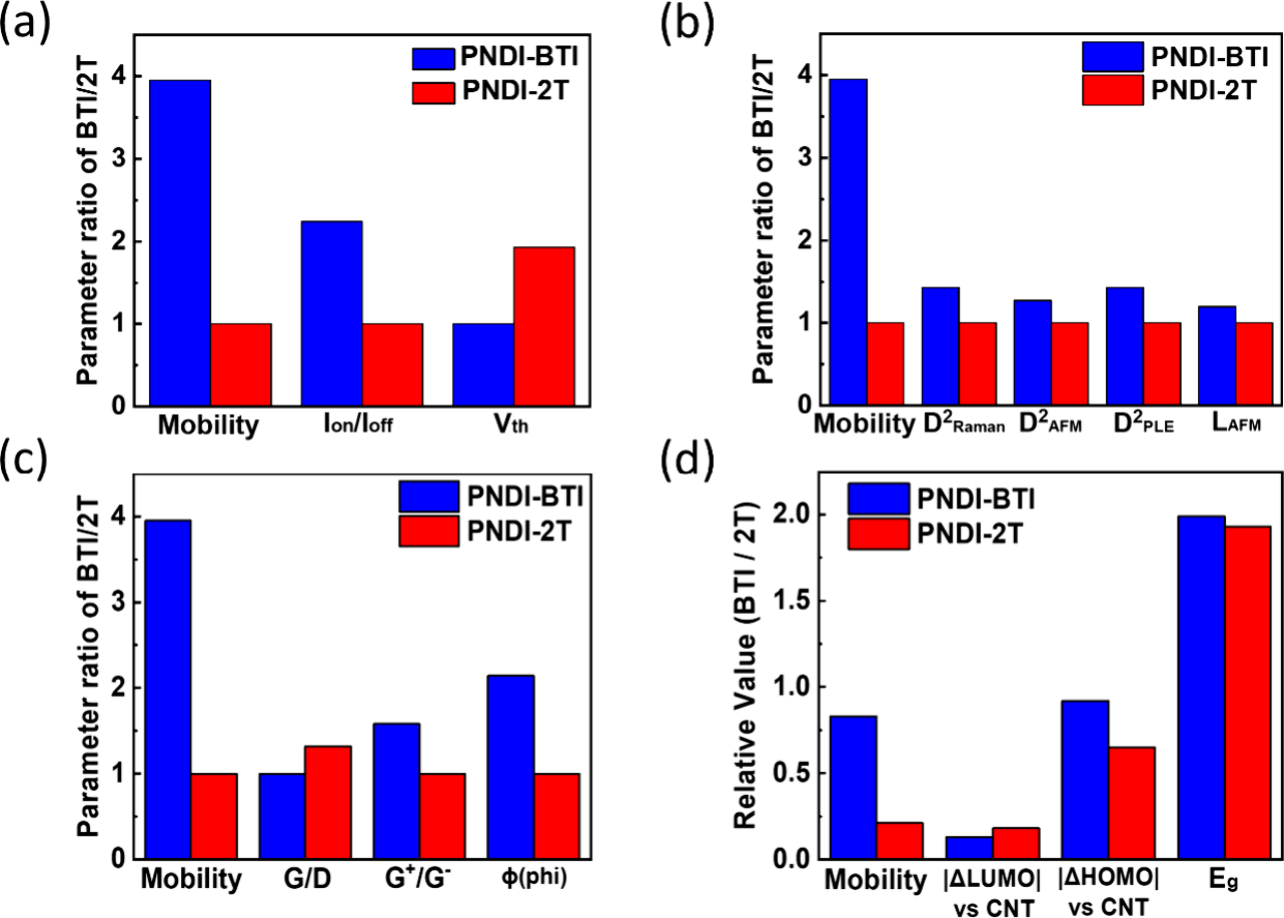
Structure–performance relationship of the conjugated polymer/s-SWCNT hybrids based on PNDI-BTI and PNDI-2T: (a) FET device parameters, (b) s-SWCNT dimensional parameters, including the diameter derived from Raman/PLE spectroscopies and AFM topography and the length from AFM topography, (c) defect and purity parameters derived from Raman and optical absorption spectroscopies, and (d) energy-level related parameters based on the HOMO and LUMO gap between the polymers and s-SWCNT and the optical bandgap of the polymers.

## Conclusion

In summary, the energy-level and coplanarity tuning of conjugated polymers play a decisive role in governing polymer–nanotube interactions, sorting selectivity, and device characteristics. The A–A type PNDI-BTI, with its larger acceptor units and reduced coplanarity, exhibited stronger aggregation and wrapping, resulting in high semiconducting purity (>99%) and longer/larger-diameter nanotubes, though at a lower yield (19%). In contrast, the D–A type PNDI-2T showed weaker aggregation and a higher yield, but compromised purity and shorter/smaller-diameter nanotube lengths. Notably, while molecular weight and dispersity of the conjugated polymers may influence polymer–nanotube sorting by affecting chain solubilization and wrapping stability,^49^ our results indicate that these factors are not the dominant determinants of selective sorting. Instead, the observed sorting trends correlate more strongly with polymer backbone conformation and energy-level alignment These contrasting behaviors directly translate into device performance. PNDI-2Se and PNDI-2Tz show poor ability to wrap around s-SWCNTs, possibly due to their unfavorable chain conformation, and therefore are excluded from subsequent characterizations of the polymer/SWCNT systems. FETs fabricated with PNDI-BTI/s-SWCNT networks achieved an *I*_on_/*I*_off_ above 9 × 10^3^ and superior operational stability, whereas PNDI-2T-based devices were limited to 2 × 10^3^ and displayed reduced uniformity. These results show that energy-level and backbone modulation not only influence aggregation tendencies but also determine the purity and morphology of nanotube networks, which, in turn, dictate transistor performance. Overall, this work highlights energy-level design as a core strategy for selective polymer-assisted sorting of s-SWCNTs. Looking ahead, further refinement of energy-level engineering will help balance purity and yield, thereby informing future directions in polymer design. These design principles are expected to accelerate the development of high-performance s-SWCNT-based electronics and to extend their applications to sensors and memory devices, thereby laying the foundation for next-generation carbon nanotube electronics.

## Experimental Section

### Materials

NDI-based polymers were synthesized and are described in the Supporting Information (Figures S1–S4). The number-averaged molecular weight (*M*_n_) and dispersity (*Đ*_M_) of the polymers are 66,200/1.69 for PNDI-2T; 10,900/1.42 for PNDI-BTI; 6,730/4.23 for PNDI-2Tz; and 26,400/8.83 for PNDI-2Se, respectively. Plasma-discharge single-walled carbon nanotubes (PD-SWCNTs, >90% carbon basis) were purchased from Sigma-Aldrich. Dextran, PMMA, phenylbis­(2,4,6-trimethylbenzoyl)­phosphine oxide (97%), and pentaerythritol tetrakis­(3-mercaptopropionate) (>95%) were obtained from Sigma-Aldrich. All chemicals and solvents were used as received without further purification.

### Selective Sorting of s-SWCNTs

The conjugated polymer (5 mg) was dissolved in toluene (20 mL) at room temperature and fully dispersed using an ultrasonic cleaner (DC300H, DELTA Ultrasonic Co., Ltd.). Subsequently, PD-SWCNTs (10 mg) were added to a polymer/SWCNT mixture with a weight ratio of 1:2. The mixture was sonicated at 40% amplitude for 30 min using a tip-type sonicator (VCX750, Sonic & Materials, Inc.). isopropanol was used in an ice bath to maintain the temperature at approximately −40 °C. The resulting dispersions were centrifuged at 12000 rpm (RCF ≈ 42,600 g) and 25 °C for 1 h using a high-speed centrifuge (FL3012, FANLINYL). Finally, the supernatant enriched with s-SWCNTs was collected for subsequent characterizations and device fabrication.

### Construction of FET Devices

Silicon wafers with a 300 nm SiO_2_ layer were cut into 1.5 × 1.5 cm^2^ pieces and cleaned in toluene, isopropanol, acetone, deionized water, and ethanol under bath sonication. After drying under a nitrogen flow, the substrates were exposed to oxygen plasma treatment for 5 min. A dextran aqueous solution (40 mg mL^–1^) was spin-cast at 4000 rpm for 60 s, followed by heating at 140 °C for 10 min to remove moisture. The treated wafer was then immersed in a diluted polymer/s-SWCNT dispersion (sorting solution: toluene = 2:1 by volume) and incubated for 3 days to allow nanotube adsorption. Unbound polymer residues were removed by rinsing with toluene several times, and the surface was further coated with PMMA (40 mg mL^–1^ in toluene) at 4000 rpm for 60 s to stabilize the nanotube layer. For substrate preparation, an SBS-based solution containing phenylbis­(2,4,6-trimethylbenzoyl) and pentaerythritol tetrakis­(3-mercaptopropionate) (25:1:1 by weight, 1.6 wt % in toluene) was spin-coated onto a fresh Si/SiO_2_ wafer (300 nm oxide). The transferred PMMA/s-SWCNT layer was placed onto the SBS-coated wafer using water. The SBS layer was photo-cross-linked by heating at 120 °C for 10 min, after which the PMMA top layer was removed by rinsing in acetone. Finally, gold electrodes (40 nm) were thermally evaporated through a shadow mask to define channels with a length (*L*) of 100 μm and a width (*W*) of 2000 μm.

### Characterizations

Optical analysis evaluated the aggregation behavior of the conjugated polymers using UV–vis–NIR absorption spectroscopy on a Jasco V-770 spectrometer over 400–1600 nm. To determine the aggregation fraction in toluene, polymer samples were prepared in both 1-chloronaphthalene (1-CN) and toluene with concentrations of 0.05 and 0.25 mg mL^–1^, respectively. The polymer dissolved in 1-CN was regarded as the disordered reference state. The fraction of aggregated species in toluene was calculated by comparing the optical absorption spectra of the two solvents. The calculation of the aggregation fraction follows the reported method.^50,51^ Raman spectra of the polymer/s-SWCNT films were measured from drop-cast samples on glass substrates using a UniDRON spectrometer (CL Technology Co., Ltd.) with 633 nm excitation. For the assignment of chirality for s-SWCNTs, photoluminescence excitation/emission analysis was performed on the solution of two polymers/PD SWNT hybrids by using the Horiba Jobin Yvon spectrofluorometer (Fluorolog-3 with Fluor Essence). The surface morphology of the polymer/s-SWCNT films was examined using an AFM100 plus (Hitachi) in tapping mode at room temperature. The obtained images were further analyzed to evaluate nanotube dispersion and film uniformity. The electrical performance of the fabricated FETs was measured using a Keithley 4200-SCS semiconductor parameter analyzer under ambient atmosphere. The effective capacitance of the gate dielectric (*C*total) was evaluated by treating the SiO_2_ and SBS layers as series capacitors, expressed as $\frac{1}{C\mathrm{total}}=\frac{1}{{C\mathrm{SiO}}_{2}}+\frac{1}{C\mathrm{SBS}}$ ,where *C*SiO2 and *C*SBS are the areal capacitances of the SiO_2_ oxide and SBS dielectric, respectively. The hole mobility (μ) and threshold voltage (*V*th) were calculated following the slope or extrapolation of the square root of drain-to-source current (*I*ds^1/2^) versus gate voltage (*V*g) in the saturation region of the transfer curves: *I*ds = $\frac{W}{2L}C_{\mathrm{total}}\mu_{h}{\left(V_{g}-V_{th}\right)}^{2}$ , where *W* and *L* are the width and length of the channel electrodes.

### Simulation

DFT calculations were performed to optimize the ground-state geometries of the conjugated polymers. The initial polymer structures were constructed with three repeating units, with long alkyl side chains replaced by methyl groups to reduce computational cost. Geometry optimization was conducted using Gaussian 09 with the B3LYP functional and a 6–31G basis set. MD simulations were subsequently performed to investigate the interaction between the conjugated polymers and SWCNTs. The procedure followed previously reported protocols. The simulations focused on polymers interacting with armchair (10, 9) semiconducting SWCNTs in a vacuum. The molecular structures of the polymers were generated and preoptimized in Avogadro using the Merck molecular force field (MMFF94s), and MD simulations were performed using the COMPASSIII force field implemented in the Materials Studio framework. Force-field-assigned charges were used to describe interatomic interactions, while long-range Coulombic interactions were treated with the Ewald summation method. van der Waals interactions were computed using an atom-based cutoff of 2 nm. The simulation process consisted of the following steps: (i) a 40 ps NVT run at 0.5 GPa to compress the system to the target density; (ii) a 100 ps NVT run at 0.0001 GPa to relax the simulation cell; (iii) annealing from 298 to 598 K for five cycles, each involving a 300 ps NVT run at 0.0001 GPa; (iv) an additional 100 ps NVT run at 0.0001 GPa to monitor density fluctuations; and (v) a final 200 ps NVT run to equilibrate the total energy and obtain the optimized configuration for subsequent property calculations. The potential energies of the nanotube (*E*_CNT_), the polymer (*E*_polymer_), and the polymer–nanotube complex (*E*_complex_) were extracted from the simulations, and the binding energy (*E*_b_) was calculated as *E*_b_ = *E*_complex_ – *E*_polymer_ – *E*_CNT_.

## Supplementary Material



## Data Availability

Data will be made available on request.

## Notes

Syntheses and chemical structure characterizations of the N-type conjugated polymers; optical and electrochemical characterization data of the polymers, including the optical absorption spectra and cyclic voltammetry profiles; tables of polymer properties, sorting parameters, and device performances; transfer characteristics of the polymer/s-SWCNT-based FETs (PDF)
