# Supplementary material for: Acceptor–Acceptor-Type Conjugated Polymers for Energy Level Modulation in Semiconducting Carbon Nanotube Transistors
Source: ACS Appl Mater Interfaces. 2025 Dec 28;18(1):2265–76. doi: 10.1021/acsami.5c21549 (PMC12781118; doi:10.1021/acsami.5c21549)
Supplement: Supplementary file 1 [file am5c21549_si_001.pdf]

Supporting Information

Acceptor–Acceptor-Type Conjugated Polymers for Energy Level Modulation in Semiconducting Carbon Nanotube Transistors

You-Chen Chen,[a] *Megumi Matsuda,*[b] *Yi-Hsuan Tung,*[a] *Guo-Hao Jiang,*[a] *Yu-Che Kan,*[a]

Shang-Wen Su,[a] *Chien-Chung Shih,*[c] *Tomoya Higashihara,*[b*] *Yan-Cheng Lin*[a,d*]

[a] Department of Chemical Engineering, National Cheng Kung University, Tainan 70101, Taiwan

[b] Department of Organic Materials Science, Graduate School of Organic Materials Science, Yamagata University, 4-3-16 Jonan, Yonezawa, Yamagata 992-8510, Japan

[c] Department of Chemical Engineering and Materials Engineering, National Yunlin University of Science and Technology, Yunlin 64002, Taiwan

[d] Advanced Research Center for Green Materials Science and Technology, National Taiwan University, Taipei 10617, Taiwan

*Corresponding author. E-mail: thigashihara@yz.yamagata-u.ac.jp (T. H.), ycl@gs.ncku.edu.tw (Y.-C. L.)

Table S1. FET dielectric layer parameters.

Layer	Thickness (nm)	Dielectric constant (εr)
SiO_2	300	3.9
SBS	30	2.4

Table S2. Optical, electrochemical, and energy levels of the polymers.

	λ_{max} (nm)[a]	HOMO (eV)[b]	LUMO (eV)[b]	E_g^{CV} (eV)[c]
PNDI-BTI	386, 528	–6.02	–3.93	1.99
PNDI-2T	382, 698	–5.75	–3.62	1.93

[a] UV–Vis absorption maximum position of the conjugated polymers dissolved in toluene. [b] Derived from the CV oxidative onset potential determined using Fc/Fc^+ as an internal potential reference. [c] HOMO and LUMO gap determined from the difference between the oxidative and reductive onsets in CV profiles. [d] Energy bandgap determined from the onset wavelength in UV-Vis spectrum.

Table S3. Summary of the sorting parameters of polymer/s-SWCNT solutions. s-SWCNT concentration of the as-sorted solutions and ϕ value determined from the UV–Vis–NIR absorption spectra.

	$C_{s\text{-SWCNTs}}$ (gL^{-1})[a]	ϕ[a]	Purity (%)	Yield (%)
PNDI-BTI	0.0206	0.47	>99	18.1
PNDI-2T	0.0556	0.22	~99	48.8

Mobility Calculation in the Saturation Regime:

The saturation condition is fully satisfied across the entire gate-voltage range in the transfer characteristics used in this study. For the test with V_d = –100 V, mobility is extracted over the V_g range –20 to –60 V. Under these conditions, the saturation inequality $|V_d| > |V_g - V_{th}|$ is always met for both polymers. The V_{th} of each polymer system and V_d condition are in **Table S4**. Therefore, all mobility values obtained at –100 V are unambiguously measured in the saturation regime. For the test with V_d = –10 V, the fitting window (V_g starts from 0 to –10 ~ –20 V) corresponds to the region where $I_d^{1/2}$–V_g displays a stable and nearly linear slope for both polymers. Using the experimentally obtained threshold voltages, the gate-voltage range for mobility extraction was carefully selected to avoid small shifts near the turn-on transition. Therefore, both calculations at V_d = –100 and –10 V follow the criterion for mobility extraction in a saturation regime. The device parameters were averaged from 16 measurement points with two independent devices per polymer system, four regions per device, and two measurement channels per region.

Table S4. Device performances of FETs comprising the polymer/s-SWCNT hybrid films.

	**PNDI-BTI**		**PNDI-2T**	
V_d	–10	–100	–10	–100
μ (cm^2 V^{-1} s^{-1})	0.83 ± 0.07	2.11 ± 0.18	0.21 ± 0.02	0.61 ± 0.05
V_{th} (V)	–3.75 ± 0.13	–6.42 ± 0.17	–7.24 ± 0.21	–11.43 ± 0.26
I_{on} (A)	1.72 × 10^{-4}	5.25 × 10^{-4}	1.38 × 10^{-5}	1.73 × 10^{-4}
I_{off} (A)	5.03 × 10^{-8}	5.81 × 10^{-8}	9.05 × 10^{-9}	6.33 × 10^{-8}
$I_{on/off}$	(3.42±0.16) × 10^3	(9.05±0.37) × 10^3	(1.53±0.12) × 10^3	(2.74 ± 0.23) × 10^3

Materials.

Palladium diacetate, tetrabutylammonium bromide, 2-bromothiazole, diisopropylethylamine, lithium diisopropylamide, trimethyltin chloride, biselenophene, *n*-butyllithium, 3,3'-dibromo-2,2'-bithiophene, acetic anhydride, 2-octyl-1-dodecanol, triphenylphosphine, *N*-bromosuccinimide, potassium phthalimide, hydrazine hydrate, 4-dimethylaminopyridine, lithium diisopropylamide, Bu_3SnCl, tris(dibenzylideneacetone)dipalladium, tri-*o*-tolylphosphine, and synthetic solvents, including chlorobenzene, chloronaphthalene, tetrahydrofuran, diethyl ether, 1,4-dioxane, and chloroform, were purchased from Tokyo Chemical Industry Co., Ltd., Luminescence Technology Corp., KANTO CHEMICAL CO., INC., FUJIFILM Wako Pure Chemical Corporation, and Sigma-Aldrich. 4,9-Dibromo-2,7-bis(2-octyldodecyl)benzo[lmn][3,8]-phenanthroline-1,3,6,8-tetraone (**NDI**),[1] 2-octyldodecan-1-amine,[1] [2,2'-bithiophene]-3,3'-dicarboxylic acid) (**2T-diacid**),[2] dithieno[3,2-c:2',3'-e]oxepine-4,6-dione,[3] and 5-(2-octyldodecyl)-4H-dithieno[3,2-c:2',3'-e]azepine-4,6(5H)-dione (BTI),[3] and 5-(2-octyldodecyl)-2,8-bis(tributylstannyl)-4*H*-dithieno[3,2-c:2',3'-e]azepine-4,6(5H)-dione (**BTI-Tin**)[4] were synthesized according to the previously reported method. The polymerization of PNDI-2T was reported in our previous work.[5] All chemicals were used as received without further purification.

Synthesis of 2Tz (2,2′-bithiazole).

A mixture of palladium diacetate (58 mg, 0.25 mmol) and tetrabutylammonium bromide (818 mg, 2.5 mmol) was prepared under a nitrogen atmosphere. A solution of 2-bromothiazole (440 μL, 5 mmol) and diisopropylethylamine (850 μL, 5 mmol) in toluene (5 mL) was added dropwise to the above mixture. The reaction mixture was refluxed for 12 hours under a nitrogen atmosphere. After cooling to room temperature, the mixture was quenched with water and extracted with ethyl acetate. The combined organic layers were washed with brine, dried over anhydrous $MgSO_4$, and concentrated under reduced pressure. The residue was purified by column chromatography on silica gel using ethyl acetate as the eluent, affording **2Tz** as an orange solid (336 mg, 40% yield). ^{1}H NMR (500 MHz, $CDCl_3$, **Figure S1a**) δ = 7.90 (*d*, *J* = 3.2 Hz, Ar-H), 7.44 (*d*, *J* = 3.1 Hz, Ar-H).

Synthesis of 2Tz-Tin (5,5′-bis(trimethylstannyl)-2,2′-bithiazole).

2Tz (220 mg, 1.3 mmol) was dissolved in dry tetrahydrofuran (4 mL) and cooled to –90 °C under argon. Freshly prepared lithium diisopropylamide (2.6 ml, 2.0 M in THF, 5.2 mmol) was added dropwise. The reaction mixture was stirred at –90 ℃ for 1 hour, and a solution of trimethyltin chloride (5.2 mL, 5.2 mmol) in THF was added dropwise at the same temperature. The mixture was then slowly warmed to room temperature and stirred overnight. After completion, the reaction was quenched with water and extracted with dichloromethane. The combined organic layers were dried over anhydrous $MgSO_4$ and concentrated under reduced pressure. The crude product was recrystallized from n-hexane to yield **2Tz-Tin** as a yellow solid (128 mg, 20% yield). ^{1}H NMR (500 MHz, $CDCl_3$, **Figure S1b**) δ = 7.80 (*t*, *J* = 7.1 Hz, Ar-H), 0.45 (*m*, Sn-CH_3).

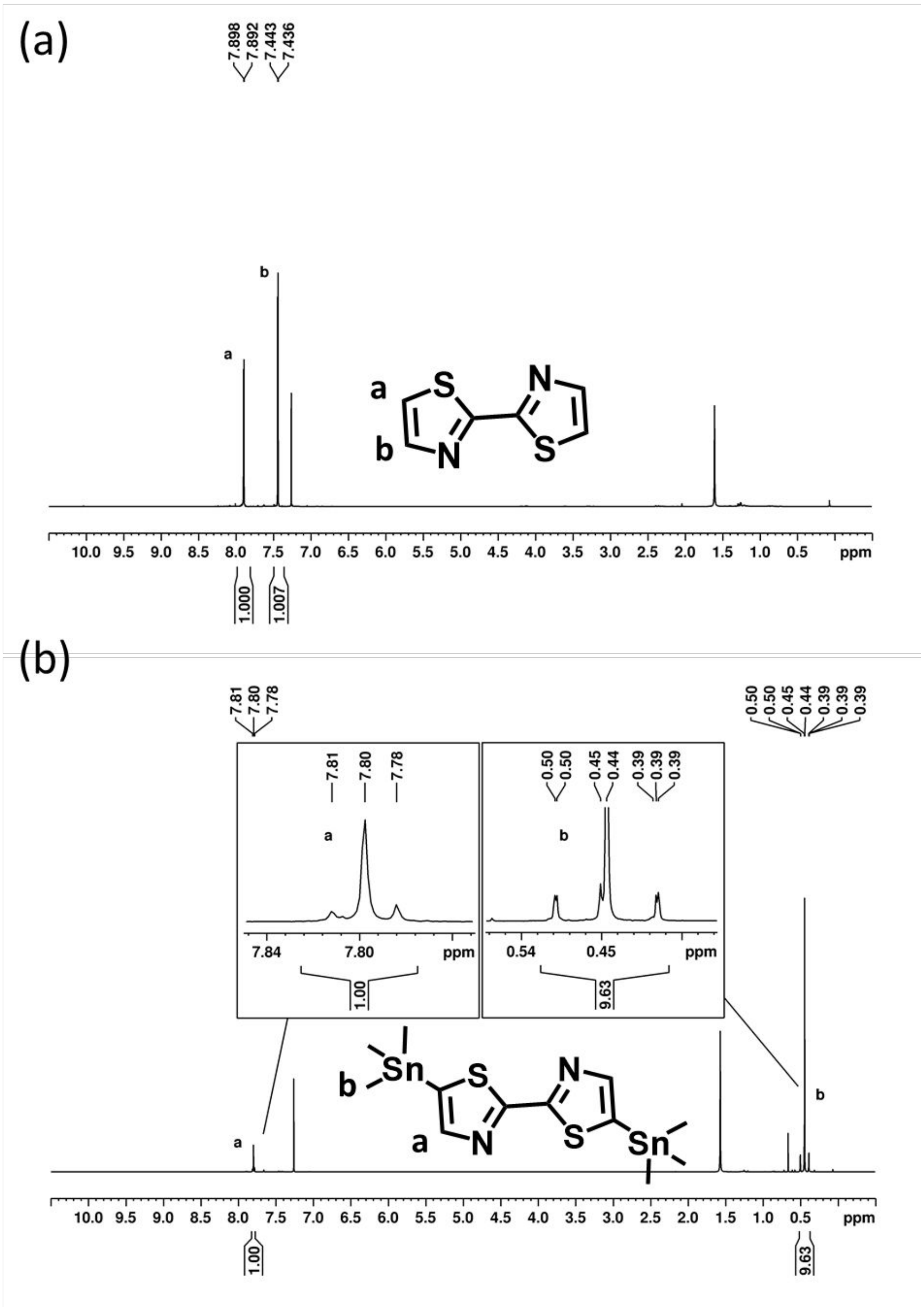

Figure S1. ^{1}H NMR spectra of (a) **2Tz** and (b) **2Tz-Tin** in $CDCl_3$.

Synthesis of 2Se-Tin (5,5′-bis(trimethylstannyl)- 2,2′-biselenophene).

2Se (biselenophene, 183 mg, 0.7 mmol) was dissolved in dry tetrahydrofuran (4.55 mL) and cooled to –78 °C under a nitrogen atmosphere. Freshly prepared n-butyllithium (0.6 mL, 1.47mmol) was added dropwise. The reaction mixture was stirred at –78 ℃ for 1 hour, and a solution of trimethyltin chloride (300 mg, 1.47 mmol) in THF (1.47 mL) was added dropwise at the same temperature. The mixture was then slowly warmed to room temperature and stirred overnight. After completion, the reaction was quenched with water and extracted with ether. The combined organic layers were dried over anhydrous $MgSO_4$ and concentrated under reduced pressure. The crude product was recrystallized from ethanol to yield **2Se-Tin** as a yellow solid (84.7 mg, yield = 21.2%). ^1H NMR (500 MHz, $CDCl_3$, **Figure S2**) δ = 7.36 (*d*, J = 5.0 Hz, Ar-H), δ = 7.34 (*d*, J = 5.0 Hz, Ar-H), 0.36 (*m*, Sn-CH_3).

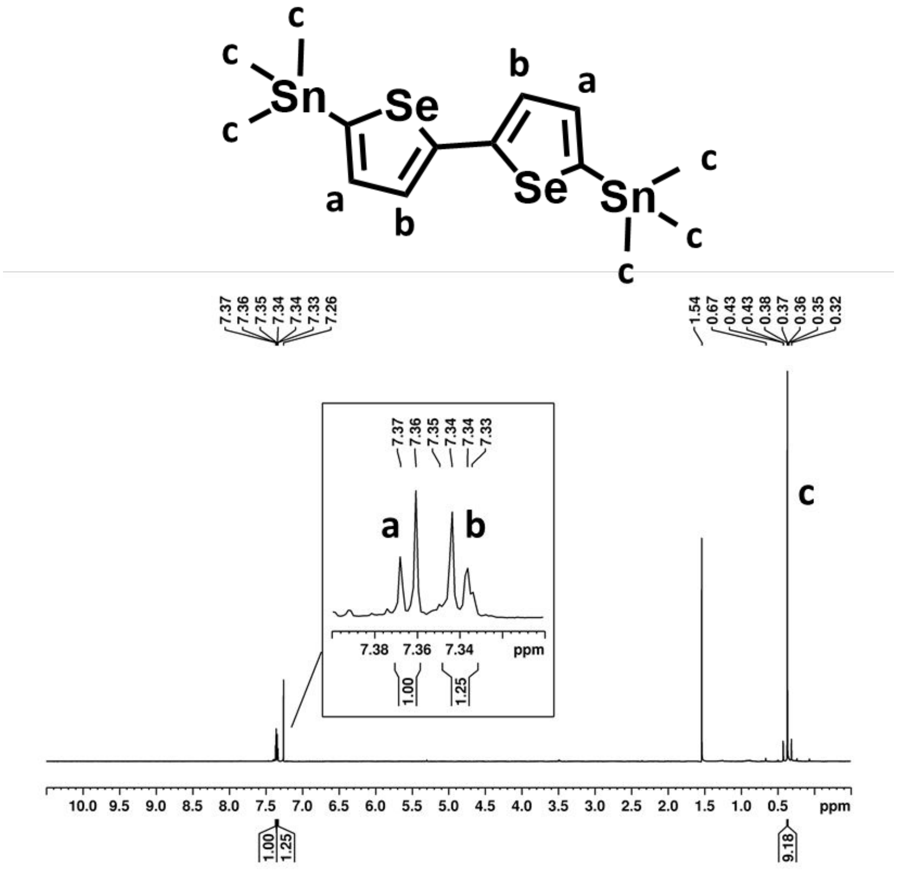

Figure S2. ^1H NMR spectra of **2Se-T** in in $CDCl_3$.

Synthesis of PNDI-2Tz.

A mixture of NDI (276 mg, 0.28 mmol), **2Tz-Tin** (138 mg, 0.28 mmol), tris(dibenzylideneacetone)dipalladium ($Pd_2(dba)_3$) (26 mg, 0.028 mmol), and tri-*o*-tolylphosphine (P(*o*-tolyl)$_3$) (86 mg, 0.28 mmol) in degassed chlorobenzene (5.6 mL) was stirred vigorously at 130 ℃ under nitrogen atmosphere for 24 hours. After cooling to room temperature, the reaction mixture was poured into methanol (300 mL) to precipitate the polymer. The solid was collected by filtration and purified by Soxhlet extraction sequentially with acetone, *n*-hexane, and chloroform. The chloroform fraction was concentrated and reprecipitated into methanol. The resulting polymer was collected by filtration and dried under vacuum overnight to afford PNDI-2Tz as a dark solid (240 mg, yield = 86%). Molecular weight evaluated by using SEC eluted by THF at 40 °C: M_n = 6,730, M_w = 28,500, $Đ_M$ = 4.23.

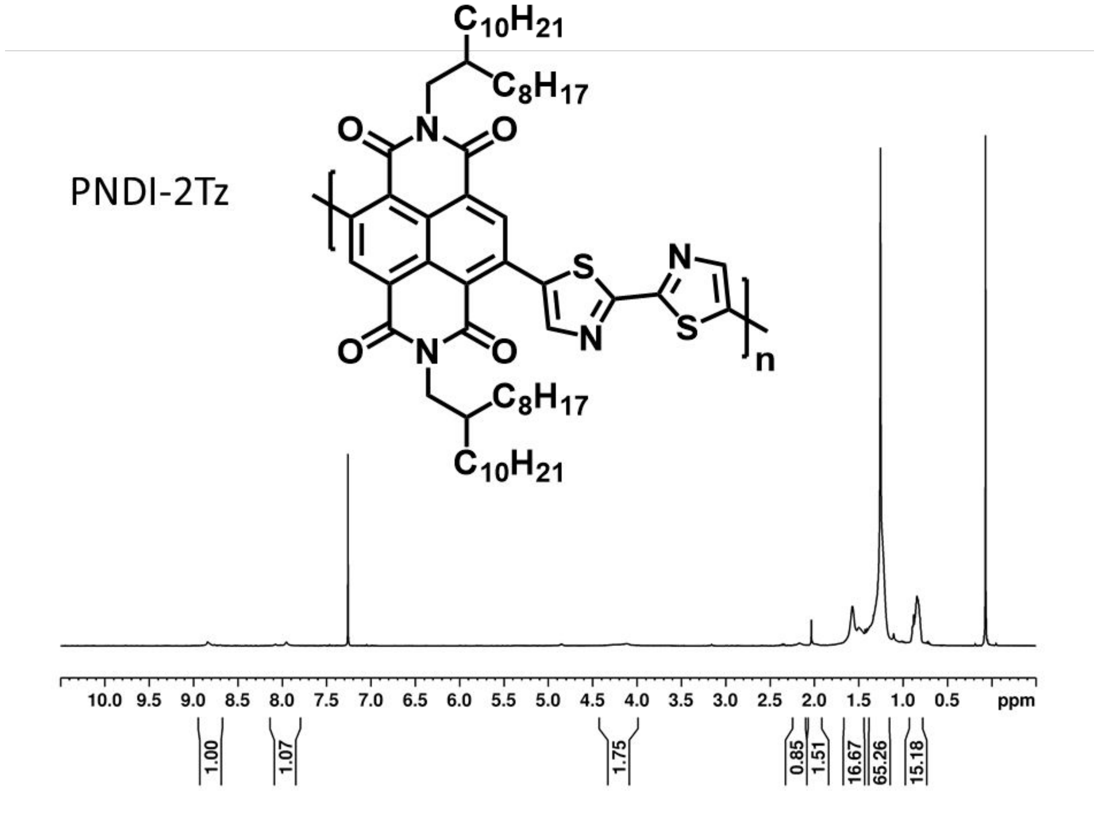

Figure S3. ^{1}H NMR spectra of PNDI-2Tz in $CDCl_3$.

Synthesis of PNDI-2Se.

A mixture of NDI (142 mg, 0.14 mmol), **2Se-Tin** (85 mg, 0.14 mmol), $Pd_2(dba)_3$ (7.69 mg, 0.0084 mmol, and P(o-tolyl)$_3$ (8.52 mL, 0.28 mmol) in degassed chlorobenzene (5.6 mL) was stirred vigorously at 130 ℃ under a nitrogen atmosphere for 24 hours. After cooling to room temperature, the reaction mixture was poured into methanol (300 mL) to precipitate the polymer. The solid was collected by filtration and purified by Soxhlet extraction sequentially with acetone, *n*-hexane, and chloroform. The chloroform fraction was concentrated and reprecipitated into methanol. The resulting polymer was collected by filtration and dried under vacuum overnight to afford PNDI-2Se as a dark solid (118 mg, yield = 76%). Molecular weight evaluated by using SEC eluted by THF at 40 ^{o}C: M_n = 26,400, M_w = 233,000, $Đ_M$ = 8.83.

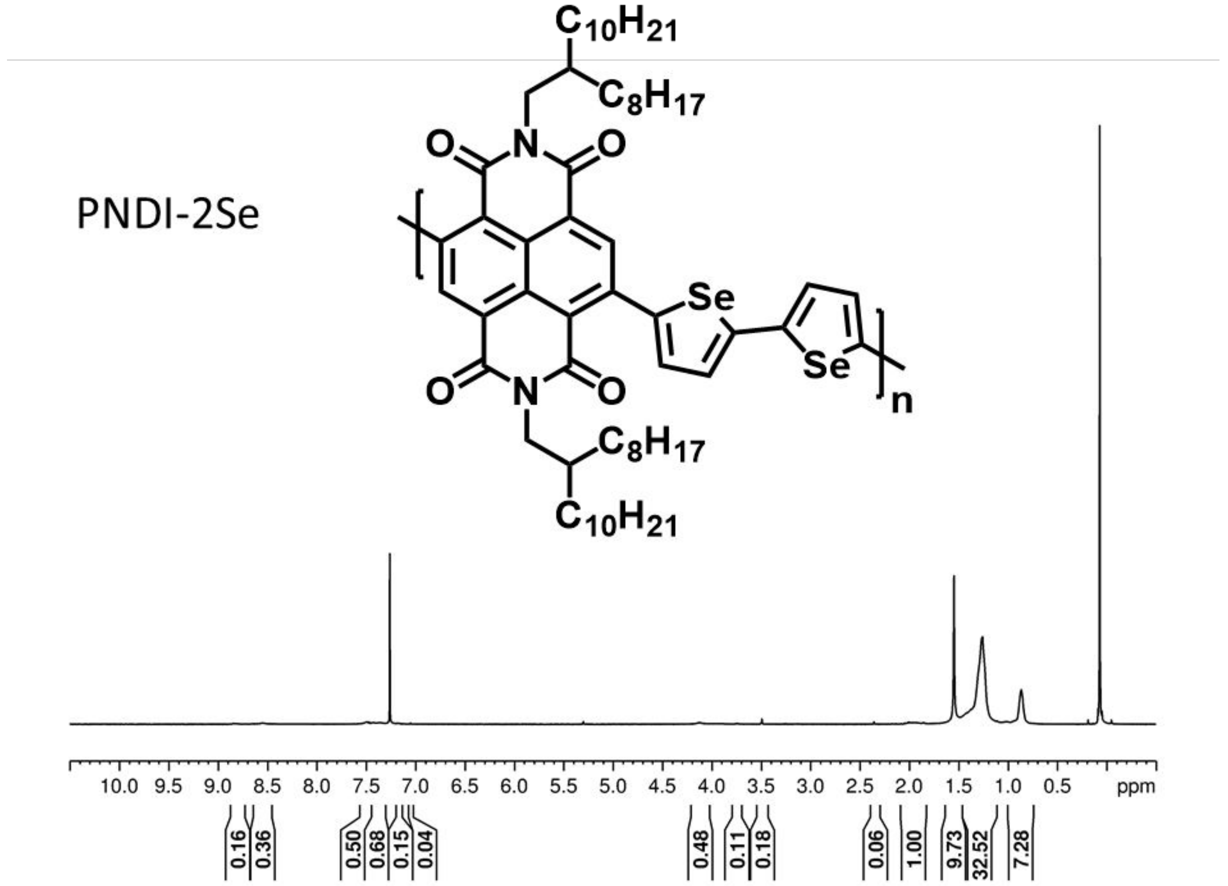

Figure S4. ^{1}H NMR spectra of PNDI-2Se in $CDCl_3$.

Synthesis of PNDI-BTI.

A mixture of **NDI** (110 mg, 0.10 mmol), **BTI-Tin** (109 mg, 0.10 mmol), $Pd_2(dba)_3CHCl_3$ (4.0 mg, 0.0039 mmol, and P(*o*-tolyl)$_3$ (5.2 mg, 0.017 mmol) in degassed chlorobenzene (8 mL) was stirred under microwave heating using a microwave synthesizer (Biotage Initiator+) following these stepwise conditions: 120 °C for 5 min, 140 °C for 5 min, 160 °C for 5 min, and 180 °C for 30 min. After the polymerization reaction, 2-(tributylstannyl)thiophene (40 μL) and 2-bromothiophene (90 μL) were added to the reaction mixture to proceed with end-capping reactions. The solution was stirred at 160 °C for 10 min using Biotage Initiator+ for each end-capping reaction. After the solution was cooled to room temperature, the crude polymer was poured into methanol to precipitate the crude polymer. The solid was collected by filtration and purified by Soxhlet extraction sequentially with methanol, acetone, and *n*-hexane. The *n*-hexane fraction was concentrated and reprecipitated into methanol. The resulting polymer was collected by filtration and dried under vacuum overnight to afford **PNDI-BTI** as a dark solid (112 mg, yield = 77%). Molecular weight evaluated by using high-temperature SEC eluted by *o*-dichlorobenzene at 140°C: M_n = 10,900, $Đ_M$ = 1.42.

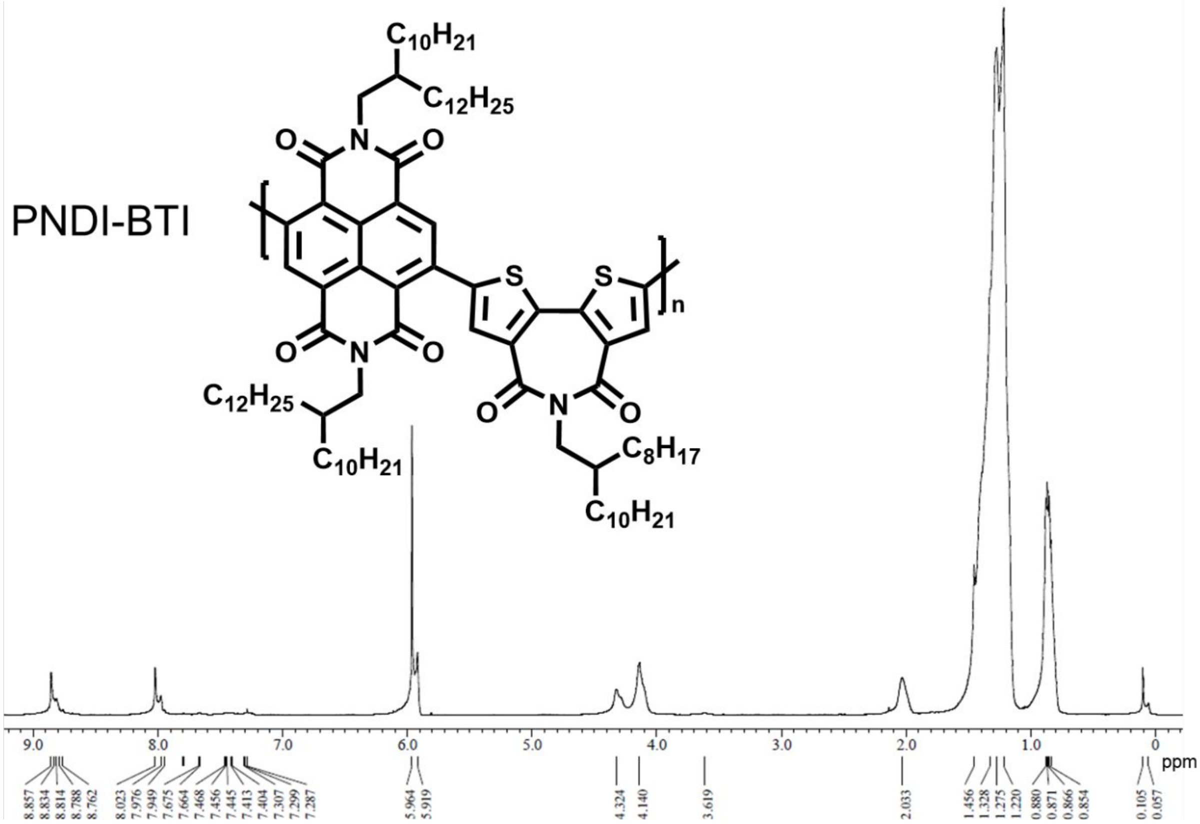

Figure S5. ^{1}H NMR spectra of PNDI-BTI in $C_2D_2Cl_4$.

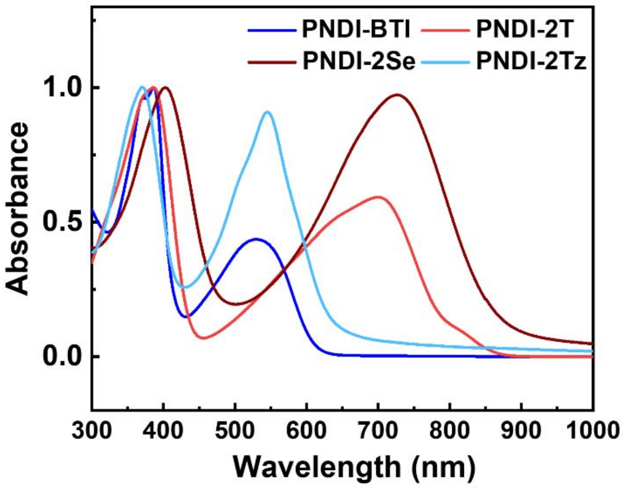

Figure S6. UV–Vis–NIR absorption spectra of the conjugated polymers PNDI-BTI, PNDI-2T, PNDI-2Se, and PNDI-2Tz measured in solution.

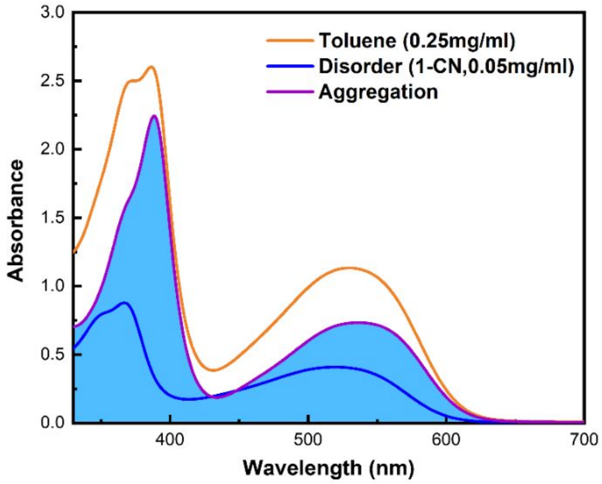

Figure S7. Aggregation and disorder fractions in the UV–Vis–NIR absorption spectra of polymers PNDI-BTI. Note that the polymer solutions in toluene were prepared at a concentration of 0.25 mg mL^{-1}, and the disordered polymer solutions were prepared in 1-chloronaphthalene (1-CN) at a concentration of 0.05 mg mL^{-1}.

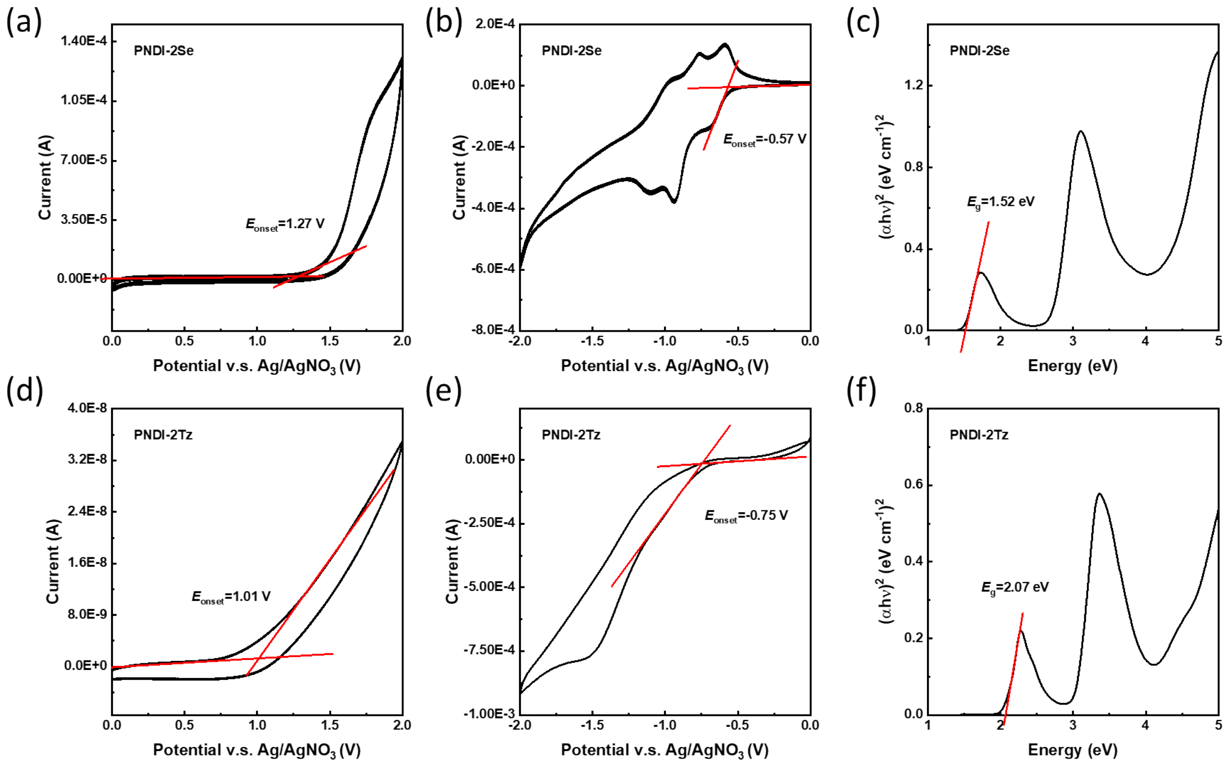

Figure S8. (a,b,d,e) CV profiles of the polymer films coated on an ITO glass. The measurement was conducted at a scanning rate of 0.1 V s^{-1}: (a,d) Oxidation profiles of and (b,e) reduction profiles of (a,b) PNDI-2Se and (d,e) PNDI-2Tz. τ-plot of the optical absorption profiles to evaluate the optical bandgap of (c) PNDI-2Se and (f) PNDI-2Tz.

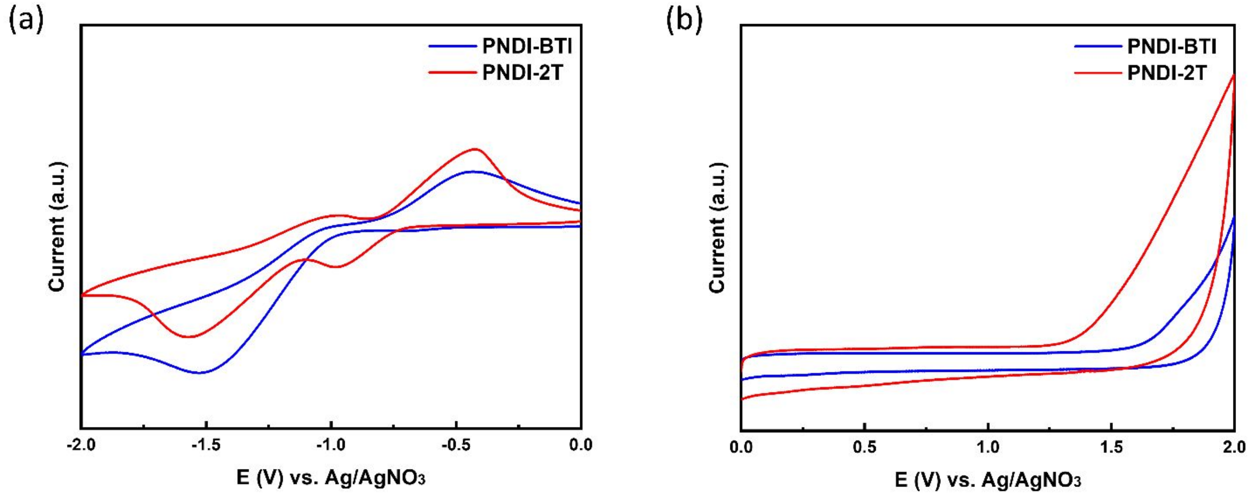

Figure S9. CV profiles of the polymer films (PNDI-BTI: blue line; PNDI-2T: red line) coated on an ITO glass. The measurement was conducted at a scanning rate of 0.1 V s^{-1}: (a) Oxidation profiles and (b) reduction profiles.

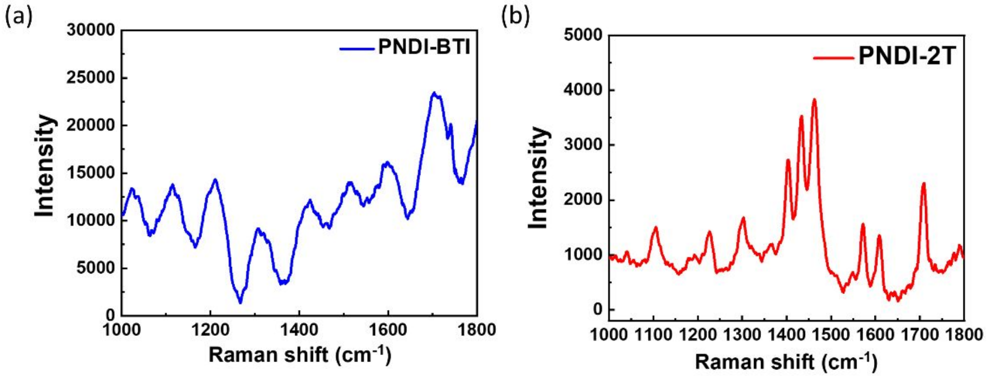

Figure S10. Raman spectra of pristine (a) PNDI-BTI and (b) PNDI-2T polymer films measured under 633 nm excitation. Note that no effective Raman signal can be observed from the PNDI-BTI film.

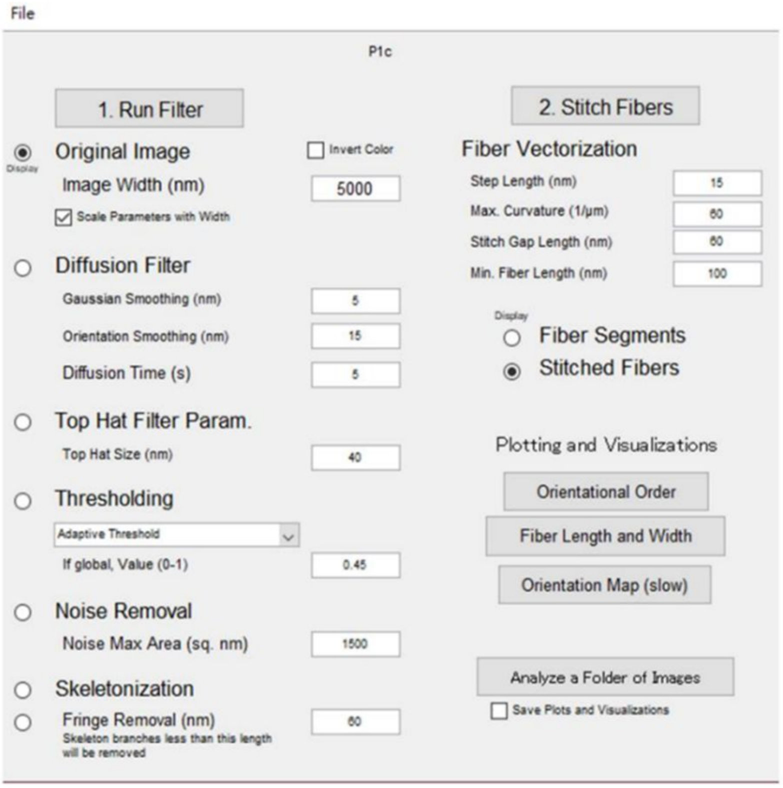

Figure S11. SWCNT morphology fitting parameters in GTFiber software for extracting the lengths and diameters from AFM topographies. The program was developed by Persson et al. and reported in *Chem. Mater.* **2017**, *29*, 3–14.

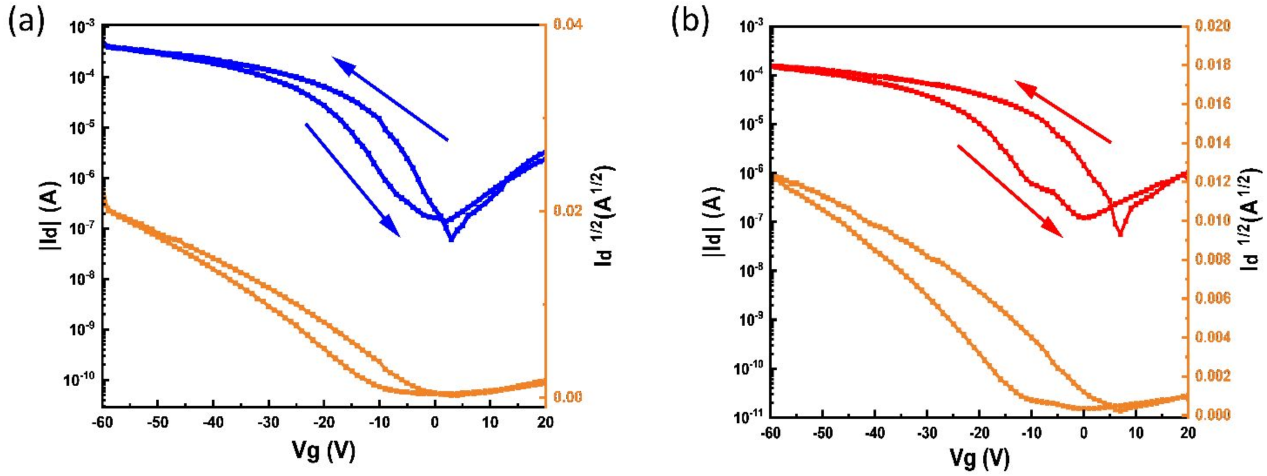

Figure S12. Transfer characteristics curves with V_d = –100 V for polymer/s-SWCNT devices of (a) PNDI-BTI/s-SWCNTs and (b) PNDI-2T/s-SWCNTs. The curves were forwardly swept from 20 to –60 V for p-type operation.

References in the Supporting Information:

(1) Guo, X.; Watson, M. D. Conjugated Polymers from Naphthalene Bisimide. *Org. Lett.* **2008**, *10* (23), 5333–5336.

(2) Wang, Y.; Guo, H.; Ling, S.; Arrechea-Marcos, I.; Wang, Y.; López Navarrete, J. T.; Ponce Ortiz, R.; Guo, X. Ladder-type Heteroarenes: Up to 15 Rings with Five Imide Groups. *Angew. Chem. Int. Ed.* **2017**, *56* (33), 9924–9929.

(3) Feng, K.; Guo, H.; Wang, J.; Shi, Y.; Wu, Z.; Su, M.; Zhang, X.; Son, J. H.; Woo, H. Y.; Guo, X. Cyano-Functionalized Bithiophene Imide-Based *n*-Type Polymer Semiconductors: Synthesis, Structure-Property Correlations, and Thermoelectric Performance. *J. Am. Chem. Soc.* **2021**, *143* (3), 1539–1552.

(4) Shi, Y.; Guo, H.; Huang, J.; Zhang, X.; Wu, Z.; Yang, K.; Zhang, Y.; Feng, K.; Woo, H. Y.; Ortiz, R.; Zhou, M.; Guo, X. Distannylated Bithiophene Imide: Enabling High-Performance *n*-Type Polymer Semiconductors with an Acceptor–Acceptor Backbone. *Angew. Chem. Int. Ed.* **2020**, *59* (34), 14449–14457.

(5) Chen, C.-C.; Su, S.-W.; Tung, Y.-H.; Wang, P.-Y.; Yu, S.-S.; Chiu, C.-C.; Shih, C.-C.; Lin, Y.-C. High-Performance Semiconducting Carbon Nanotube Transistors Using Naphthalene Diimide-Based Polymers with Biaxially Extended Conjugated Side Chains. *ACS Appl. Mater. Interfaces* **2024**, *16* (34), 45275–45288.
